# Unique Role of Caffeine Compared to Other Methylxanthines (Theobromine, Theophylline, Pentoxifylline, Propentofylline) in Regulation of AD Relevant Genes in Neuroblastoma SH-SY5Y Wild Type Cells

**DOI:** 10.3390/ijms21239015

**Published:** 2020-11-27

**Authors:** Daniel Janitschke, Anna A. Lauer, Cornel M. Bachmann, Martin Seyfried, Heike S. Grimm, Tobias Hartmann, Marcus O. W. Grimm

**Affiliations:** 1 Experimental Neurology, Saarland University, 66421 Homburg/Saar, Germany; Daniel.Janitschke@uks.eu (D.J.); Anna.Lauer@uks.eu (A.A.L.); Manuel.Bachmann@uks.eu (C.M.B.); Martin.Seyfried@uks.eu (M.S.); Heike.Grimm@uks.eu (H.S.G.); Tobias.Hartmann@uks.eu (T.H.); 2 Deutsches Institut für DemenzPrävention (DIDP), Saarland University, 66421 Homburg/Saar, Germany

**Keywords:** methylxanthines, caffeine, theobromine, theophylline, pentoxifylline, propentofylline, oxidative stress, lipid homeostasis, energy metabolism, signal transduction

## Abstract

Methylxanthines are a group of substances derived from the purine base xanthine with a methyl group at the nitrogen on position 3 and different residues at the nitrogen on position 1 and 7. They are widely consumed in nutrition and used as pharmaceuticals. Here we investigate the transcriptional regulation of 83 genes linked to Alzheimer’s disease in the presence of five methylxanthines, including the most prominent naturally occurring methylxanthines—caffeine, theophylline and theobromine—and the synthetic methylxanthines pentoxifylline and propentofylline. Methylxanthine-regulated genes were found in pathways involved in processes including oxidative stress, lipid homeostasis, signal transduction, transcriptional regulation, as well as pathways involved in neuronal function. Interestingly, multivariate analysis revealed different or inverse effects on gene regulation for caffeine compared to the other methylxanthines, which was further substantiated by multiple comparison analysis, pointing out a distinct role for caffeine in gene regulation. Our results not only underline the beneficial effects of methylxanthines in the regulation of genes in neuroblastoma wild-type cells linked to neurodegenerative diseases in general, but also demonstrate that individual methylxanthines like caffeine mediate unique or inverse expression patterns. This suggests that the replacement of single methylxanthines by others could result in unexpected effects, which could not be anticipated by the comparison to other substances in this substance class.

## 1. Introduction

**Figure 1:**
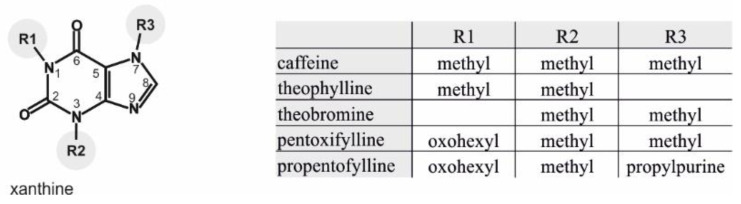
Chemical structure of xanthine (**left**) and combinations of the side chains of its derivatives caffeine, theophylline, theobromine, pentoxifylline and propentofylline (**right**).

Methylxanthines are derivatives of the purine base xanthine (2,6-dihydroxypurine) with additional methyl groups at the nitrogen of the xanthine structure (R1, R2, R3) (Figure 1). The naturally occurring methylxanthine caffeine is characterized by three additional methyl groups (1,3,7-trimethylxanthine), whereas theophylline and theobromine have two additional methyl groups at different positions (theophylline = 1,3-dimethylxanthine; theobromine = 3,7-dimethylxanthine). Synthetic methylxanthines used as pharmaceuticals have, in addition to the methyl group at the R2 position, other functional groups at the R1 and R3 positions. For example, pentoxifylline has, in addition to the methyl groups at R2 and R3, an oxohexyl group at R1 (3,7-dimethyl-1-(5-oxohexyl)purin-2,6-dion), and propentofylline has a propylpurine group instead of a methyl group at R3 (3-methyl-1-(5-oxohexyl)-7-propylpurine-2,6-dione). 

The naturally occurring methylxanthines caffeine, theophylline and theobromine are widely consumed all over the world, being present in coffee, green and black tea and cacao [1,2]. A total of 81.2% of the participants in a study investigating time trends of non-alcoholic beverage intake among German citizens declared a frequent consumption of coffee [3]. Coffee and green or black tea represent the second most commonly consumed non-alcoholic beverages, after water [4]. Methylxanthines can be easily absorbed from the digestive tract after oral intake and are known to have psychostimulant actions [5,6].

Aside from dietary uptake, xanthine derivatives are or have been widely used pharmacologically in veterinary and human medicine. For example, methylxanthines are reported to be beneficial for patients with chronic obstructive lung disease (COPD) and asthma by attenuating bronchial spasms [7] and for the treatment of respiratory pediatric diseases [8]. Since methylxanthines can cross the blood–brain barrier and influence the central nervous system via their effect as adenosine receptor antagonists, they are reported to have beneficial properties in respect of neuronal and neurodegenerative disorders like schizophrenia, Parkinson’s disease and Alzheimer’s disease (AD) [9,10,11]. In respect to coronary heart diseases, methylxanthines are reported to prevent hypertension, arterial thrombosis and myocardial infarction, as they have been shown to affect platelet function and arterial vasodilation [7,12]. In addition to their beneficial effects in treating the side effects of radiotherapy, individual methylxanthines are reported to be useful for biochemical modulation of cancer chemotherapy [13].

Methylxanthines act via different mechanisms, including antagonism of purinergic P1 receptors, mainly adenosine A1 and A2A receptors [14]. Blocking adenosine receptors reduces inhibitory endogenous adenosine and modulates several cellular pathways, e.g., by increasing dopaminergic activity in the brain and thereby mediating the psychostimulant effect. In higher concentrations, which cannot be reached by nutritional intake, but rather are reached through pharmaceutical intake, methylxanthines lead to inhibition of cyclic nucleotide phosphodiesterases and high-affinity ATP-dependent cyclic nucleotide transporters, resulting in an increase of cellular cyclic AMP levels and mobilization of intracellular calcium; as a consequence a modulation of gamma-amino butyric acid receptors, for example, has been reported [15,16,17,18].

Methylxanthines affect gene transcription via different mechanisms. Naturally occurring methylxanthines effectively bind to all base pairs of native double helical DNA in a dose-dependent manner via hydrogen bond interaction [19]. Moreover, it has been shown that caffeine and theophylline are able to suppress the replication of human immunodeficiency virus type I (HIV-1) by inhibiting specific kinases that are important for retroviral DNA integration [20]. Theophylline used in therapeutic concentrations increases the activity of histone deacetylases. Regulating the histone deacetylases further results in the regulation of the transcription of inflammatory genes, for example, which has been reported in epithelial cells and macrophages [21]. However, the exact underlying molecular mechanism has yet to be elucidated [22]. Another potential mechanism of methylxanthines in regulating gene expression seems to include adenosine receptors. Methylxanthines bind to these receptors, which have a G-protein-dependent influence on the adenylate cyclase-mediated conversion of adenosine triphosphate (ATP) to cyclic adenosine monophosphate (cAMP). cAMP acts as an allosteric effector for protein kinase A, which phosphorylates transcription factors like the cAMP response element-binding protein (CREB) [23]. Interestingly, impairments in CREB signaling, which is important for neuronal plasticity and the formation of long-term memory, can be linked to pathological conditions occurring in neurodegenerative diseases like AD [24]. In addition to these mechanisms of action, including adenosine receptors, direct interaction with DNA and modulation of histone deacetylases, methylxanthines might also act directly, for example, as antioxidants.

**Figure 2:**
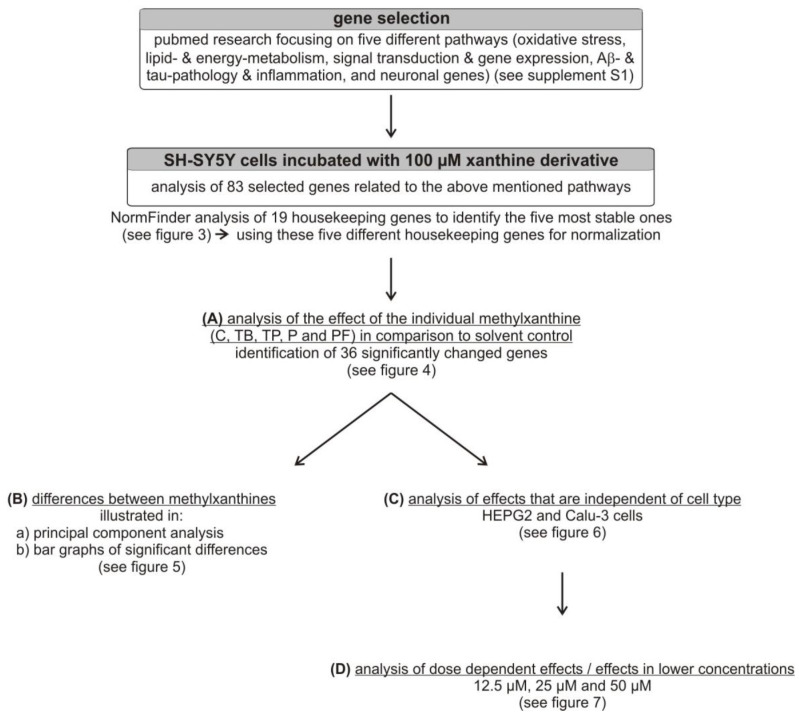
Design of the study. C: caffeine, TB: theobromine, TP: theophylline, P: pentoxifylline, PF: propentofylline.

In summary, methylxanthines are widely consumed and have a broad scope of application in diseases, including neurodegenerative diseases like AD, Parkinson’s disease, Huntington’s disease and multiple sclerosis. However, little is known about whether structural changes between the xanthine derivatives caffeine, theobromine, theophylline, pentoxifylline and propentofylline may mediate a unique function. In the present study, we therefore addressed the question of whether these natural and synthetic methylxanthines exert different effects in respect to their transcriptional properties on the expression of genes in neuroblastoma SH-SY5Y wild-type cells involved in pathways related to neurodegenerative disorders, e.g., oxidative stress, lipid and energy metabolism, signal transduction and gene expression, or Aβ- and tau-pathology and inflammation. The design of the study is summarized in Figure 2. Eighty-three genes were selected that are known to be linked to neurodegenerative diseases. Transcriptional changes induced by each analyzed xanthine derivative in comparison to the solvent control were examined (A). Thereupon, we addressed the question of whether there is a difference between the single methylxanthines, utilizing multiple comparison analysis (B). To elucidate if these observed effects are specific for neuronal cells or are cell-type-independent, more cell lines (HEPG2–liver and Calu-3–lung) (C) and different concentrations were analyzed (D).

## 2. Results

### 2.1. Selection of Stable Housekeeping Genes

**Figure 3:**
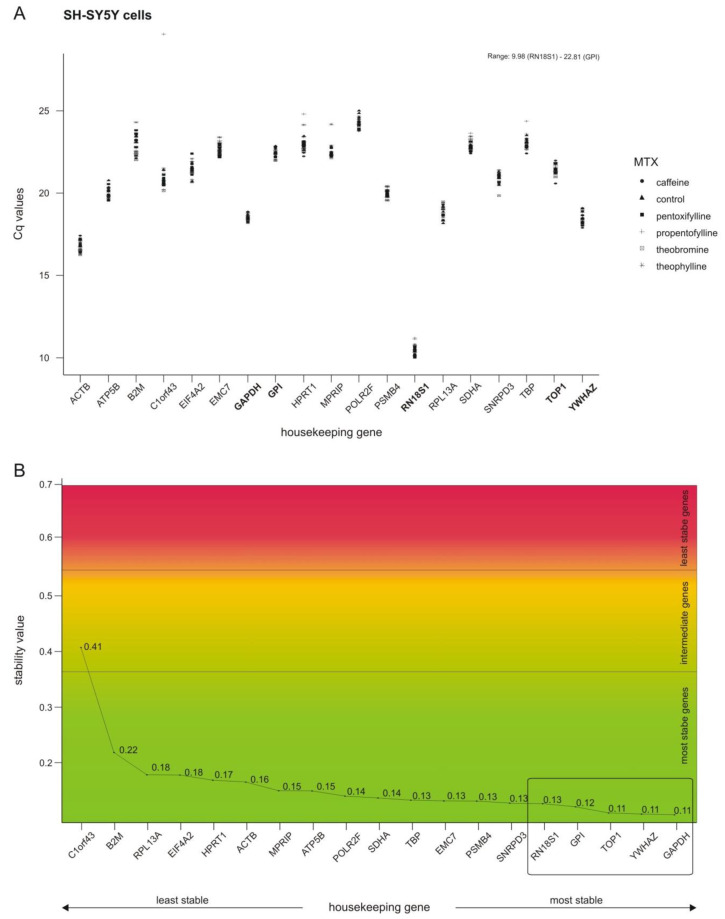
Selection of housekeeping genes (HKGs) for normalization in SH-SY5Y cells. (**A**) Expression levels of the selected putative HKGs given as quantitative real-time RT-PCR cycle threshold (Cq) values in SH-SY5Y cells after treatment with solvent control, caffeine, theophylline, pentoxifylline, theobromine or propentofylline. The five most stable HKGs are presented in bold letters. (**B**) Expression stability values for 19 analyzed genes calculated by the NormFinder algorithm. Classification of stability values in most stable genes (0 < stability value < 0.37; green), intermediate genes (0.37 < stability value < 0.53; yellow) and least stable genes (0.53 < stability value < 0.69; red) was done in accordance with Penna et al. [25]. HKGs are ranked from the least to the most stable from left to right and the five most stable genes selected for normalization in SH-SY5Y cells (*RN18S1*, *GPI*, *TOP1*, *YWHAZ* and *GAPDH*) are highlighted by a box.

In accordance with the ∆∆CT method, quantitative real-time polymerase chain reaction (RT-PCR) results are normalized to a housekeeping gene (HKG). One indispensable characteristic of the selected housekeeping gene has to be a constant expression, which is independent of the investigated parameter. To evaluate which housekeeping gene was not influenced by methylxanthines in this underlying study, 19 different genes, commonly used in the literature as HKGs [25,26,27], were selected. As well as a stable expression, the HKG should have a comparable expression level to that of the gene being analyzed, or, if several genes of interest are being analyzed, should cover a broad range of expression levels. In line with this second requirement, the Cq values of the selected HKGs varied from 9.98 (*RN18S1*) to 22.81 (*GPI*) (see Figure 3A).

NormFinder was used to calculate a stability value for each gene, which enables a ranking of the analyzed genes according to their stability (see Figure 3B). Based on these NormFinder results and the expression level, we selected the five most stable HKGs covering a broad range of expression for SH-SY5Y cells: *GAPDH*, *GPI*, *RN18S1*, *TOP1* and *YWHAZ*. Importantly, these chosen HKGs are linked to different cellular pathways (carbohydrate metabolism, glucose metabolism, protein biosynthesis, transcription and signal transduction). For further analysis, the average Cq values of the best five selected HKGs were used, making it even less likely that observed alterations were due to coincidental changes in a single HKG. Additional information about the NormFinder results, and the results leading to the selection of the HKGs utilized for the cell lines Calu-3 and HEPG2 are shown in Supplementary File S2.

### 2.2. Transcriptional Effects of Caffeine, Theobromine, Theophylline, Pentoxifylline and Propentofylline in Neuronal Cells

As described above, methylxanthines can influence gene expression via several different mechanisms, including receptor-mediated pathways, effect on DNA methylation or direct interaction with DNA. Eighty-three genes were selected that are linked to neurodegenerative diseases including pathways associated with oxidative stress, lipid- and energy-metabolism, signal transduction and gene expression, Aβ- and tau-pathology and inflammation, as well as neuronal genes. The rationale for selecting these genes and their potential link to neurodegenerative and other diseases are summarized in Supplementary File S1. Interestingly, several of these pathways also affect diseases like coronary heart disease, lung and liver diseases, which are also reported to be influenced by methylxanthines [7,8,28]. Here we evaluated the effect on the expression of genes involved in transversal biological processes, especially genes linked to the five pathways listed in Figure 2. Potential alterations in gene expression caused by methylxanthines found in this study are therefore mostly not specific to neurodegenerative diseases but have to be considered as more common mechanisms relevant to a variety of diseases.

To address the question of whether methylxanthines influence the gene expression of the abovementioned selected genes and pathways, human neuroblastoma cells (SH-SY5Y) were incubated for 24 h with 100 µM of the xanthine derivatives or water as a solvent control. As methylxanthines, caffeine (C), theobromine (TB), theophylline (TP), pentoxifylline (P) and propentofylline (PF) were chosen. These substances reflect the most widely consumed methylxanthines as nutrients (C, TP, TB) or are synthetic methylxanthines used as a leading structure for further modifications (P, PF).

The use of 100 µM is slightly above the maximal plasma concentration observed in humans (8–10 mg/L, approx. 50 µM [29]), however it has to be taken into consideration that caffeine is consumed frequently over years and the experimental cell culture setting is a short 24 h incubation, which might be the cause for widely using this concentration in other studies, showing no cytotoxicity and not influencing cellular proliferation [11]. Moreover, it has to be mentioned that the maximum plasma concentration of these compounds might be higher after pharmaceutical interventions than after consumption as a nutrients in a diet.

**Figure 4:**
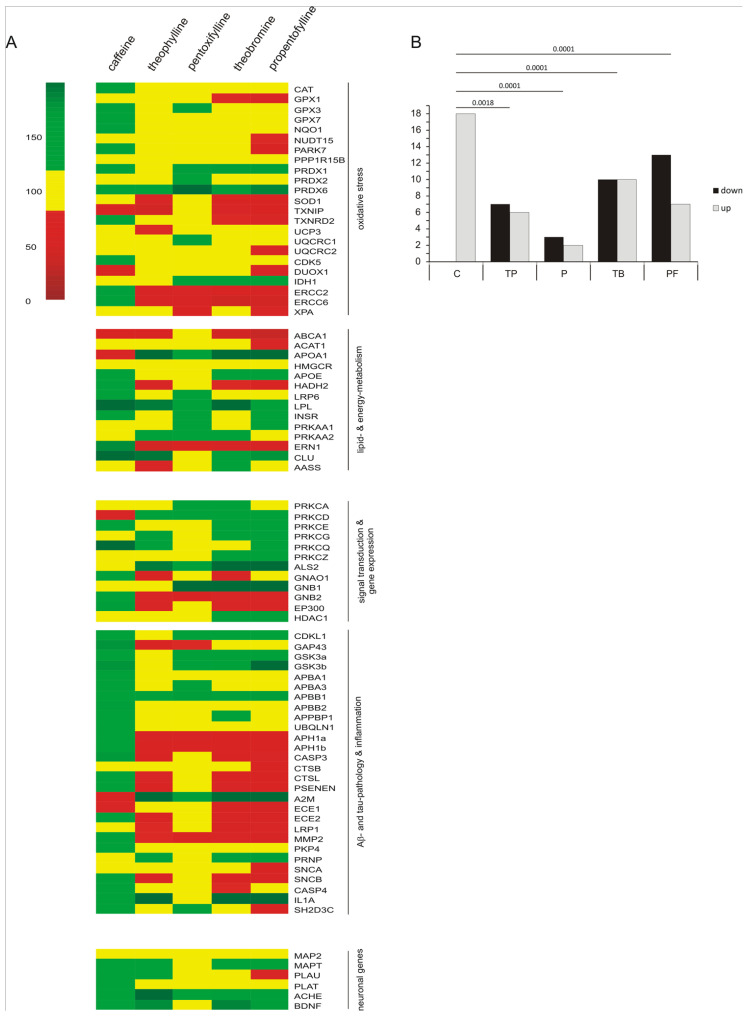
Transcriptional influence of caffeine, theobromine, theophylline, pentoxifylline and propentofylline in SH-SY5Y cells. (**A**) Transcriptional changes of genes related to the different pathways involved in oxidative stress, lipid and energy metabolism, signal transduction and gene expression, and Aβ- and tau-pathology and inflammation after treatment of human neuroblastoma cells (SH-SY5Y) for 24 h with 100 µM of the analyzed xanthine derivatives, in comparison to the solvent control, are illustrated in a heatmap. Fold changes greater than the standard deviation (yellow) are highlighted in red when downregulated and in green when upregulated. (**B**) The number of genes which are significantly changed by a single methylxanthine are summarized in a bar diagram. To calculate the significance of the observed effects we first performed an ANOVA for each gene to examine if there are differences in general. Afterwards, these *p*-values were adjusted with the false discovery rate method over all 83 analyzed genes. To determine which methylxanthine significantly changed the expression of a gene compared to the solvent control, we performed Dunnett’s test. To examine if there are significant differences in the distribution patterns of the significantly down- or upregulated genes influenced by the analyzed methylxanthines, we performed Fisher’s exact test.

After extraction of RNA and reverse transcription, RT-PCR was performed to analyze changes in gene expression. Results are summarized in the heatmap shown in Figure 4A and a list of individual effect strenghts, standard errors of the mean and *p*-values for each methylxanthine and gene can be found in Supplementary File S3. The number of genes significantly up- or downregulated by each analyzed methylxanthine is illustrated as a bar diagram next to the heatmap (Figure 4B). To analyze the pattern, Fisher’s exact test was utilized, revealing that there are significant changes in the distribution of down- and upregulated genes, especially for caffeine compared to the other methylxanthines, suggesting that caffeine has unique, opposed or different effects compared to the other methylxanthines.

In detail, caffeine significantly increased the expression of three oxidative stress-related genes (*PARK7*, *TXNRD2*, *ERCC2*), whereas pentoxifylline, theobromine and propentofylline upregulated the expression of two genes in this pathway (*PRDX1*, *PRDX6*). Propentofylline had the highest downregulating effect on oxidative stress-related genes by significantly reducing the expression of four different genes compared to the solvent control (*PARK7*, *SOD1*, *TXNRD2*, *ERCC2*). The expression of two and three genes, respectively, were decreased by theophylline and theobromine in this pathway (TP: *TXNIP*, *ERCC2*; TB: *SOD1*, *ERCC2*, *ERCC6*). In respect to lipid and energy metabolism, caffeine and theobromine showed the most upregulating properties by influencing the transcription of three genes respectively (C: *HADH2*, *LPL*, *ERN1*; TB: *APOA1*, *LPL*, *AASS*). Moreover, the expression of *APOA1* was significantly increased after treatment with pentoxifylline and propentofylline. Theobromine and propentofylline had an additional reducing influence on the expression of genes related to lipid and energy metabolism by significantly decreasing the transcription of *ABCA1* (TB and PF) and *HADH2* (PF). Three genes involved in pathways linked to signal transduction and gene expression were significantly upregulated by caffeine treatment (*PRKCQ*, *GNB2*, *EP300*), whereas theobromine, theophylline and propentofylline increased the expression of *ALS2*. In contrast to the increasing impact of caffeine on *GNB2* and *EP300* expression, those genes were downregulated by theophylline, theobromine, pentoxifylline and propentofylline. In respect to genes related to Aβ- and tau-pathology and inflammation, caffeine had the most prominent upregulating effect by mediating a significant increase in the transcription of *GAP43*, *APBB2*, *UBQLN1*, *APH1b*, *CASP3*, *PSENEN*, *ECE2* and *CASP4*. The expression of three genes involved in these pathways was increased by theophylline, theobromine and propentofylline (*A2M*, *PRNP*, *IL1A*). Moreover, propentofylline has the most pronounced downregulating effect by influencing the transcription of five genes involved in Aβ- and tau-pathology and inflammation (*APH1a*, *APH1b*, *PSENEN*, *ECE2*, *MMP2*), followed by theobromine (*APH1a*, *APH1b*, *ECE2*, *LRP1*), theophylline (*APH1a*, *APH1b*, *ECE2*) and pentoxifylline (*APH1a*, *MMP2*). The following neuronal genes were significantly upregulated: *PLAU* by caffeine, *ACHE* by theophylline and *MAPT* by theobromine (see Figure 4).

### 2.3. Comparison of Caffeine, Theobromine, Theophylline, Pentoxifylline and Propentofylline in Respect to their Transcription-Regulatory Effects

**Figure 5:**
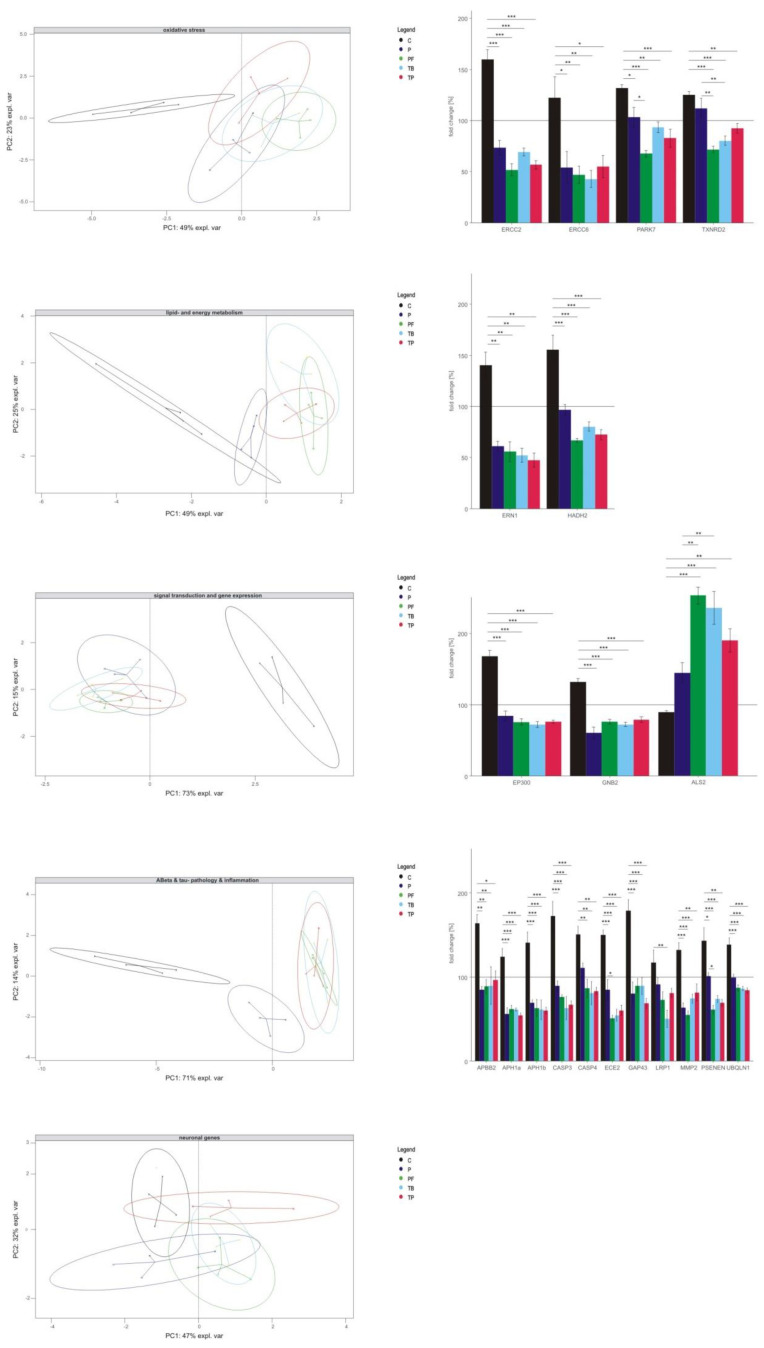
Comparison of the transcriptional effects of caffeine, theobromine, theophylline, pentoxifylline and propentofylline. Results of the principal component analysis for each analyzed pathway are shown on the left. Fold changes and significances of those genes mediating the trends seen in the principal component analysis are illustrated in a bar diagram on the right for each pathway. Standard error of the mean is shown by the error bars and significance was calculated by Tukey’s honestly significant difference (HSD) post-hoc test and set as * *p* ≤ 0.05, ** *p* ≤ 0.01 and *** *p* ≤ 0.001.

Interestingly, the results described above reveal no uniform expression pattern between the single methylxanthines, indicating the greatest difference between caffeine and the other xanthine derivatives. By performing a principal component analysis (PCA) for each pathway, we were able to exemplify how caffeine, theobromine, theophylline, pentoxifylline and propentofylline relate to each other in this examined pathway (Figure 5, left). PCA serves to structure, simplify and illustrate a multivariate data set by converting it into a two-dimensional data set consisting of two main components which make up the largest percentage of variance in the multivariate dataset, in which component one (*x*-axis) is more important than component two (*y*-axis). The genes which are included in these two components can be found respectively in the loading plot for each pathway (see Supplementary File S6). For oxidative stress, lipid and energy metabolism, signal transduction and gene expression, Aβ- and tau-pathology and inflammation the PCA clearly showed a separation of caffeine from the other analyzed methylxanthines in component one. In the group of neuronal genes, no clear separation in component one could be observed, whereas a separation in component two between pentoxifylline and theophylline can be found (for detailed information see Supplementary File S6).

To further clarify whether the separation of caffeine from the other xanthine derivatives, as indicated in the PCA, was significant, we performed multiple comparison analysis with Tukey HSD post-hoc test (individual *p* values are listed in Supplementary File S4). In regard to oxidative stress caffeine regulates the expression of *ERCC2*, *ERCC6*, *PARK7*, and *TXNRD2* significantly inversely in comparison to theobromine, theophylline, pentoxifylline and propentofylline (see Figure 5, right bar diagram). A similar result was obtained for genes related to lipid and energy metabolism. Examples of the exclusive effect of caffeine are the expression of the genes *ERN1* and *HADH2*. Analyzing genes involved in signal transduction and gene expression, principal component analysis provides strong evidences for a unique function of caffeine compared to the other xanthine derivatives. This becomes even more obvious when the single genes are examined individually because three out of four significantly changed genes in this pathway were contrarily regulated by caffeine compared to the other methylxanthines (*EP300*, *GNB2* and *ALS2*). In respect to Aβ- and tau-pathology and inflammation a similar exclusive influence of caffeine became apparent. The expression of twelve out of 14 genes was regulated in an opposite way by caffeine compared to the other analyzed xanthine derivatives (*APBB2*, *APH1a*, *APH1b*, *CASP3*, *CASP4*, *ECE2*, *GAP43*, *LRP1*, *MMP2*, *PSENEN* and *UBQLN1*).

### 2.4. Analysis of Cell Type- and Dose-Dependence of the Observerd Methylxanthine-Mediated Effects

**Figure 6:**
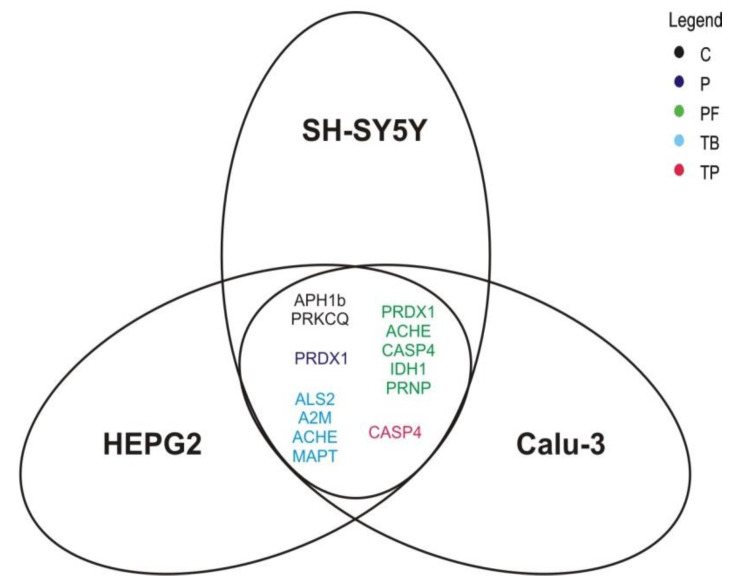
Analysis of methylxanthine-mediated transcriptional effects, independent of cell type. The Venn diagram illustrates the genes of which the expressional changes, mediated by the analyzed methylxanthines (shown in different colors), are conserved over the three cell lines SH-SY5Y, HEPG2 and Calu-3. C: caffeine, TB: theobromine, TP: theophylline, P: pentoxifylline, PF: propentofylline.

As mentioned above, the selected genes are only partially specific for neurodegenerative diseases but are also affected in a variety of other diseases due to their common mechanisms. Therefore it is reasonable to determine whether the observed effects are specific for neuronal cells or are also present in other cell lines. We therefore analyzed Calu-3 and HEPG2 cells to identify gene regulatory effects that are not limited to neuroblastoma SH-SY5Y cells. Calu-3 and HEPG2 cells were incubated under comparable conditions (24 h duration and 100 µM concentration) and analyzed for overlapping effects in the expression of those genes which were identified to be changed significantly compared to the solvent control. The Venn diagram in Figure 6 illustrates those genes which showed a significant effect calculated over all three cell lines. A detailed overview of the transcriptional changes is presented in Supplementary File S5. Caffeine significantly upregulates the expression of *APH1b* (125.0% ± 9.4%, *p* = 0.035) and *PRKCQ* (166.5% ± 52.6%, *p* = 0.029), whereas theobromine significantly increases the transcription of *ALS2* (161.1% ± 40.1%, *p* = 0.045), *A2M* (294.1% ± 126.2%, *p* = 0.008), *ACHE* (175.0% ± 31.1%, *p* = 0.002) and *MAPT* (153.7% ± 14.5%, *p* = 0.036) over all three analyzed cell lines. Theophylline significantly downregulates the expression of *CASP4* (74.9% ± 5.0%, *p* = 0.017) and pentoxifylline upregulates that of *PRDX1* (141.9% ± 16.7%, *p* = 0.025) in a significant way, though it is conserved in SH-SY5Y, HEPG2 and Calu-3 cells. The synthetic xanthine derivative propentofylline increases the transcription of *PRDX1* (148.6% ± 24.9%, *p* = 0.007), *ACHE* (161.7% ± 22.9%, *p* = 0.016), *IDH1* (141.7% ± 22.6%, *p* = 0.026) and *PRNP* (133.6% ± 17.8%, *p* = 0.041) and reduces the expression of *CASP4* (78.1% ± 9.1%, *p* = 0.046) independently of the cell type.

**Figure 7:**
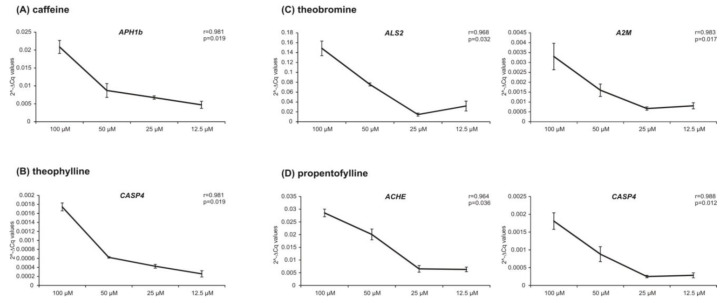
Analysis of dose-dependent effects of methylxanthines. The expression of *APH1b*, *ALS2*, *A2M*, *CASP4*, *ACHE* and *CASP4* are plotted against the concentrations of caffeine (**A**), theophylline (**B**), theobromine (**C**) and propentofylline (**D**) in diagrams, respectively. The Pearson correlation coefficient *r* and the corresponding *p*-value are shown for each gene.

Furthermore, we were interested in whether the alterations in the expressions are dose-dependent effects of the analyzed methylxanthines. To examine effects in lower concentrations, we incubated SH-SY5Y cells for 24 h additionally with 12.5 µM, 25 µM or 50 µM of caffeine, theobromine, theophylline, pentoxifylline or propentofylline. Those genes of which the expression was influenced in a similar way by xanthine derivatives in all three analyzed cell lines were analyzed. By calculating the correlation between housekeeping genes’ normalized Cq-values and the four different methylxanthine concentrations, we found six genes to be regulated in a dose-dependent manner. The results for those genes, namely *APH1b*, *CASP4*, *ALS2*, *A2M*, and *ACHE*, are shown in Figure 7. The expression of the genes examined further did not correlate significantly with the analyzed methylxanthine concentrations (caffeine: *PRKCQ*: *r* = 0.378, *p* = 0.622; pentoxifylline: *PRDX1*: *r* = 0.880, *p* = 0.120; theobromine: *ACHE*: *r* = 0.943, *p* = 0.057; *MAPT*: *r* = 0.735, *p* = 0.256; propentofylline: *PRDX1*: *r* = 0.903, *p* = 0.097; *IDH1*: *r* = 0.856, *p* = 0.144; *PRNP*: *r* = 0.761, *p* = 0.239).

## 3. Discussion

Numerous beneficial effects of naturally occurring and synthetic methylxanthines are known. This study was performed to clarify the influence of the methylated xanthine derivatives caffeine, theobromine, theophylline, pentoxifylline and propentofylline on the expression of selected genes and to detect potential differences between the methylxanthines. Our findings suggest that methylxanthines are highly linked with oxidative stress, lipid and energy homeostasis, signal transduction and gene expression, and pathways involved in neuronal function, since the expression of several genes related to these pathways is altered due to incubation with methylxanthines, whereas the magnitude of effect on target genes varies between the single analyzed xanthine derivatives.

Significant differences in the distribution of significantly regulated genes exist between caffeine and the methylxanthines theobromine, theophylline, pentoxifylline and propentofylline. Moreover, caffeine is the only xanthine derivative that had exclusively increasing transcriptional influences on genes compared to the solvent control in each analyzed pathway. To further evaluate this indicated trend for a unique function of caffeine, we performed principal component analysis combined with multiple comparison analysis to examine if the methylxanthine-mediated effects on gene expression differ significantly between the analyzed xanthine derivatives. In addition to common transcriptional regulations of all methylxanthines, our study reveals that specific gene regulatory effects dependent on the individual methylxanthine species, most of all caffeine, also exist.

A possible explanation could be a more potent binding of caffeine to adenosine receptors, but referring to the inhibition constant (Ki-value) or IC_50_ values for A2A receptor it becomes obvious that those values of theophylline, for example, are smaller than those of caffeine (Ki: 6.7 µM for theophylline and 9.5 µM for caffeine [30]; IC_50_: 45 µM for theophylline and 98 µM for caffeine [1]). Therefore, our findings indicate the existence of an additional, adenosine-receptor-independent mechanism by which caffeine can influence gene expression. 

Interestingly, it has been reported that caffeine itself has a beneficial effect or directly influences enzymes in lower concentrations, whereas at higher concentrations its adenosine-receptor-mediated effect is activated, resulting in gene regulatory effects which might contribute to the observed dose dependency observed even at a concentration range below the IC_50_ of caffeine. In regard to oxidative stress, it has been reported that methylxanthines have an antioxidative property by reducing the reactive oxygen species (ROS) level itself. Moreover, an interaction of superoxide dismutase enzyme (SOD) with caffeine was recently reported, resulting in a superimposed antioxidant activity and suggesting SOD as an effective carrier of caffeine [31]. Another reason for the individual effects of the methylxanthines might be a direct interaction with transcriptional regulators. Such an interaction has been described for theophylline, which is able to restore corticosteroid responsiveness in COPD and asthma patients through direct activation of the histone deacetylase (HDAC) [32].

A further reason for this exclusive function of caffeine compared to the other analyzed methylxanthines could be the structural substitutions of the xanthine molecule. For numerous physiologic effects, different pharmacological potencies for the xanthine derivatives caffeine, theobromine and theophylline were reviewed by Monteiro and colleagues (2016). They summarized that caffeine is effective in the stimulation of the central nervous system, whereas theophylline, for example, is more effective in coronary dilatation. Additionally, the generally less active naturally occurring methylxanthine theobromine is considered to be a potent cardiac stimulant. Moreover, they assigned the substitutions in different positions of the xanthine molecule to affinity and selectivity towards adenosine receptor sides and variant physiologic effects [33]. In line with this, several pathways are described in the literature to be only or inversely influenced by caffeine in comparison to other methylxanthines. In preclinical studies, caffeine showed a more pronounced effect on the intracellular calcium concentration in ventricular myocytes compared to the other naturally occurring methylxanthines theophylline and theobromine [34]. Furthermore, caffeine is administrated in infants with bronchopulmonary dysplasia due to its beneficial antioxidative effects [35,36].

To investigate the global transcriptional effects of methylated xanthines, we analyzed the expression of genes of which the changes differ significantly between the incubated methylxanthines in the liver cell line HEPG2 and the lung cancer cell line Calu-3. Examining effects which are independent of the incubated cell type, we found that numerous genes associated with neuronal disorders were altered by methylxanthines. The enzyme acetylcholinesterase (AChE), encoded by the gene *ACHE*, hydrolyzes the neurotransmitter acetylcholine into choline and acetate, terminating neurotransmission [37]. In regard to methylated xanthines, it was shown that caffeine can inhibit the activity of AChE and is therefore linked to a neuroprotective effect in neurodegenerative diseases like AD [38]. *APH1b* encodes a protein which is a functional component of the γ-secretase complex involved in the cleavage of numerous substrates, for example amyloid-precursor-protein (APP) or notch. In a previous study we showed that the activity of γ-secretase is influenced in the presence of methylxanthines [11]. An additional effect of methylated xanthines on AD might be due to their transcriptionally effect on *A2M*, a gene that encodes alpha-2-macroglobulin, known to be involved in Aβ homeostasis [39]. Moreover, we found the expression of *MAPT* to be influenced by methylxanthines. *MAPT* encodes the microtubule-associated protein tau (MAPT), which is reported to be influenced by caffeine [40]. The gene *PRNP* encodes a membrane glycosylphosphatidylinositol-anchored glycoprotein, the major prion protein PrP, of which the misfolded version is linked to numerous diseases [41]. It is reported that caffeine is able to protect human neuronal cells against prion-mediated neurotoxicity due to inducement of autophagy signals [42]. Regarding to inflammation, the protein caspase 4, which is encoded by *CASP4*, is important for the execution phase of cell apoptosis and is an inducer of apoptosis when overexpressed. A link between the presence of caffeine and a change in *CASP4* expression is described in the literature [43]. The guanine exchange factor ALS2, which is encoded by *ALS2*, as well as the protein kinase C theta, encoded by *PRKCQ*, are proteins involved in signal transduction, which is known to be influenced by caffeine and other methylated xanthines [44,45]. The cytoplasmic enzyme NADP^+^-dependent isocitrate dehydrogenase 1 (IDH1) plays a crucial role in NADPH production and is therefore important for the regeneration of reduced glutathione. Therefore, the activity of this dehydrogenase can be correlated with cellular redox state since it is a key enzyme in cellular defense against oxidative stress [46]. As these pathways are affected in many diseases, the broad beneficial effects of methylxanthines can be explained. Our findings indicate a beneficial influence of methylated xanthines on neuronal disorders and are in line with reports that the frequent consumption of caffeine is linked with a lower incidence of Alzheimer’s disease and Parkinson’s disease [47,48]. A study analyzing the influence of caffeine on cholesterol-induced sporadic AD pathology, including levels of the neurotoxic Aβ protein, the phosphorylation of tau and the generation of oxidative stress, showed that a daily diet containing caffeine reversed the effects of the cholesterol-enriched diet and suggested caffeine to be protective against sporadic AD-like pathology [49]. Interestingly, we found global transcriptional effects of the analyzed methylxanthines on pathways involved in processes like APP processing, defense against oxidative stress and inflammation, which could explain the beneficial influence of xanthine derivatives on AD pathology and suggest their use for AD prevention and therapy. Since the analyzed naturally occurring and synthetic methylxanthines also have individual effects on specific genes, which might be helpful to address specific issues, these are especially important in individualized medical approaches and should be a subject for further studies. Since we found different transcriptional effects with a dependence on the concentration used, it has to be mentioned that it is important to distinguish between concentrations that can be reached by nutritional intake and higher concentrations of methylxanthines that can be reached only by pharmaceutical supplementation.

Based on our data, caffeine should be taken into consideration as a potential therapeutic approach in different diseases, which are linked to the analyzed pathways and investigated genes, in particular in Alzheimer’s disease, because this xanthine derivative shows several exclusive transcriptional effects that could be beneficial for special patterns. However, it has to be mentioned that the results obtained in this study are derived from cell culture experiments and have to be verified in vivo. Moreover, we have evaluated whether methylxanthines alter gene expression, utilizing neuroblastoma wild-type cells. In these cells, genes were altered which are known to be affected in neurodegenerative diseases in particular Alzheimer’s disease. However, since we investigated the effects of methylxanthines on gene expression in wild-type cells, further experiments have to confirm whether methylxanthines can revert or rescue neurodegenerative disease-mediated changes in gene expression, e.g., by investigating effects of methylxanthines in Alzheimer’s disease models or clinical studies. Interestingly, besides this caveat, we could confirm cell-type independent regulation for most robustly changed genes which also showed a dose-dependent regulation.

## 4. Materials and Methods

### 4.1. Chemicals and Reagents

Caffeine, theophylline, TRizol Reagent, a High-Capacity cDNA Reverse Transcription Kit and HPLC-grade H_2_O were acquired from Thermo Fisher Scientific (Waltham, MA, USA). Theobromine, pentoxifylline, propentofylline and all other chemicals were purchased from Merck, formerly Sigma-Aldrich (Darmstadt, Germany). The primers used in this study were synthesized from Eurofins MWG Operon, Eberberg, Germany.

### 4.2. Cell Culture

The human neuroblastoma cell line SH-SY5Y wild-type was cultivated in Dulbecco’s modified Eagle’s medium (DMEM) with 10% fetal calf serum (FCS, GE Healthcare Life Sciences, Chalfont St. Giles, United Kingdom) and 1% non-essential amino acid solution at 37 °C and 5% CO_2_. For cultivation of the liver hepatocellular carcinoma cell line HEPG2 wildtype, 1% penicillin/streptomycin was additionally added to the medium. The lung cancer cell line Calu-3 wild-type was cultivated in Dulbecco’s modified Eagle’s medium–F12 (DMEM F12) with 10% fetal calf serum (FCS, GE Healthcare Life Sciences, Chalfont St. Giles, United Kingdom) at 37 °C and 5% CO_2_. All cells were seeded out on six-well plates uniformly and cultivated to a confluency of 90%–100% before starting treatment with methylxanthines.

### 4.3. Cell Treatment with Methylxanthines

Sixteen hours prior to incubation, the FCS content in the DMEM cultivation medium was lowered to 0.1% FCS to preclude possible effects of the lipids present in FCS on the expression of fatty acid metabolism related genes (for example). Caffeine, theobromine, theophylline, pentoxifylline and propentofylline were incubated for 24 h (8 + 16 h) in a concentration of 100 μM, 50 μM, 25 μM or 12.5 μM and treatment with HPLC-grade H_2_O was performed as a solvent control.

### 4.4. Analysis of Gene Expression by RT-PCR Experiments

Quantitative real-time polymerase chain reaction (RT-PCR) experiments were performed after isolation of total cellular RNA using the TRIzol reagent, according to the manufacturer’s guidelines. The purity and concentration of the isolated RNA were analyzed by measuring their absorbance using a NanoDrop2000 (Thermo Fisher Scientific, Waltham, Massachusetts, USA). To reduce the influence of possible RNA contaminations, only samples with a 260/280 and 260/230 ratio ≥ 1.8 were used. Two micrograms of RNA were reverse-transcribed to generate complementary DNA (cDNA) using the High-Capacity cDNA Reverse Transcription Kit as described by the manufacturer. RT-PCR was performed with the Fast SYBR green Master Mix (Applied Biosystems, Foster City, USA) and the primers listed in Table 1 on a PikoReal Real-Time PCR System (Thermo Fisher Scientific, Waltham, Massachusetts, USA). To preclude differences in RT efficiency, the five most stable housekeeping genes (see Section 2.1, Selection of Stable Housekeeping Genes) were used for normalization.

### 4.5. Stability Analysis of Housekeeping Genes via NormFinder Algorithm

To calculate the stability value for each housekeeping gene, we used the NormFinder algorithm, published in [53] (https://moma.dk/normfinder-software), according to the authors’ manual via the given R function.

### 4.6. Data Analysis

All quantified data represent an average of four independent incubations. In accordance with the ΔΔCT method, ΔCq values of each sample were calculated by subtracting the Cq value of each single housekeeping gene from the Cq value of the gene of interest. This was performed for each housekeeping gene, respectively. Afterwards, the ΔCq values of each sample were transformed via the 2^−ΔCq^ method and those values were averaged for the five housekeeping genes. Therefore, for every gene of interest the normalization was done identically with the same housekeeping genes. Each incubation was performed four independent times, which were analyzed in four independent real-time PCRs, resulting in four independent 2^−ΔCq^ values which were used to calculate the mean and standard deviation. Fold changes were calculated using these 2^−ΔCq^ values in relation to the solvent control, which was set as 100 percent.

To calculate significant differences between the methylxanthine incubations and the solvent control, analysis of variances (ANOVA) was performed. Obtained *p*-values were adjusted via the false discovery rate (FDR) for all 83 genes to control type I errors. After a significant ANOVA, we performed Dunnett’s test post hoc to investigate if significant differences to the solvent control existed for each methylxanthine. Creation of the heatmap was performed with the help of the package “pheatmap” (Raivo Kolde 2019; https://CRAN.R-project.org/package=pheatmap). To calculate significant differences in the distribution of up- and downregulated significant genes for each methylxanthine, we carried out Fisher’s exact test and *p*-values were adjusted via FDR.

Principal component analysis was carried out for each pathway, including genes which had at least one significant change from a methylxanthine to the control. Loading plots showing the correlation coefficients for each gene influencing component one and component two can be found in Supplementary File S6. Additional post hoc analysis for each pathway was carried out similarly to the earlier method, using Tukey’s honestly significant difference (HSD) test. Computation of statistical significance and principle component analysis [54] were performed in R (R Core Team 2020; Vienna, Austria; https://www.R-project.org/). Error bar graphs represent standard error of the mean. Correlation coefficients for dose-dependent effects were calculated via the Pearson method. Significance was set at * *p* ≤ 0.05, ** *p* ≤ 0.01 and *** *p* ≤ 0.001.

## Figures and Tables

**Table 1:** RT-PCR primers used.

Gene	Primer Forward	Primer Reverse
*A2M*	GCCAACAAGGTGGATTTGAG	AGAGCTCAGCATCAGGCTTC
*AASS*	CTGTGGTGAGAGATGCAGTGA	TGCCCATTGAAAGTGACTGA
*ABCA1*	TATGAGGGCCAGATCACCTC	TGCTCATCTCAGAGCGAATG
*ACAT1*	AAATTCATATGGGCAGCTGTG	GCTTCCCATGCTGCTTTACT
*ACHE*	CACTGGTGGGAATGACACAG	ATCTACCACAGGCACGAAGG
*ACTB*	CTTCCTGGGCATGGAGTC	AGCACTGTGTTGGCGTACAG
*ALS2*	ATACCCTGACACCCAAGCAG	GGTACCTGAGATGGCACTGG
*APBA1*	CAGGAAGAAGGCTCCTGAAG	GGGTGGTCCATCATTGTCTC
*APBA3*	CATCTCCTACACAGCCGACA	GATGAGCTGGGCGTCCTC
*APBB1*	TTTGGAAGGATGAACCCAGT	AAGCTTCTCCTCCTCTTGGG
*APBB2*	GACCCAGAAGCCAAGTGTTT	GGAAAGTTGCCTGATGCAGT
*APH1a*	CAGCCATTATCCTGCTCCAT	GGAATGTCAGTCCCGATGTC
*APH1b*	GTGTCAGCCCAGACCTTCAT	CAGGCAGAGTTTCAGGCTTC
*APOA1*	CAGCTAAACCTAAAGCTCCTTGA	CTCAGGCCCTCTGTCTCCTT
*APOE*	GCAGACCGAGTGGCAGAG	CATCAGCGCCCTCAGTTC
*APPBP1/NAE1*	TCACCAAACAGACTCCATCATT	TTGCCTGAATCTGCAATCATA
*ATP5B*	GCAGGAAAGAATTACCACTACCAAG	TGGTAGCATCCAAATGGGCAA
*B2M*	TGCTGTCTCCATGTTTGATGTATCT	TCTCTGCTCCCCACCTCTAAGT [26]
*BDNF*	TACTTTGGTTGCATGAAGGC	GCCAATGATGTCAAGCCTCT
*C1orf43*	AGCTCTGGATGCCATTCGTACC	GTGTTTCGCAGATCCAGCAGGT
*CASP3*	AAGCACTGGAATGACATCTCG	ATCACGCATCAATTCCACAA
*CASP4*	GGAATGGAGCTGACTTTGACA	CTGACTCCATATCCCTGGCT
*CAT*	ATTCGATCTCACCAAGGTTTG	CTTGGGTCGAAGGCTATCTG
*CDK5*	TGTGACCAGGACCTGAAGAA	TAGCACATTGCGGCTATGAC
*CDKL1*	AGGGACCAAAGGGAATAACC	CACCCAACGATGAGTGTTTG
*CLU*	CAGCCCTTCCTTGAGATGAT	CGTCGCCTTCTCGTATGAAT
*CSTB*	CAGGACAAGCACTACGGATACA	CACAGAGAAAGCTCCCTCCA
*CTSL*	CCTGTGAAGAATCAGGGTCAG	GCCCAGAGCAGTCTACCAGA
*DUOX1*	CTGGACATCCTGGTGGTCTT	ATCCTTGGAAATGAGGCCAT
*ECE1*	TGATGATCAAGGACGGGAGT	TCTACCATGCACTCGGTCTG
*ECE2*	ACCTGATCTGGAACCTGGTG	CTCGGCACACAGGACTTCTT
*EIF4A2*	TGCTCTGCATGGTGACATGGAC	ATCCCGCGAGCCAACAAGTC
*EMC7*	GTCAGACTGCCCTATCCTCTCC	CATGTCAGGATCACTTGTGTTGAC [50]
*EP300*	TATGCCAACAGCAGCTCAAC	GGCTTGGACGAGTTTGTGA
*EPX*	GGCGTTGACTGTGAGAGGAC	GGAAGAAAGGGATGCAGTCA
*ERCC2*	GAGCCCTTTGACGACAGAAC	TGACAGACTGGAAACGCTCA
*ERCC6*	CAGCGGTTAAGGAGATGGAA	AAACCTTCGTCAAATTCAGCA
*ERN1*	GTAATCTCTGAGGGCAGCCA	GTCTTCGTGCTTCTCTGGGT
*GAPDH*	ATTCCACCCATGGCAAATTC	GGGATTTCCATTGATGACAAGC
*GAP43*	AGAGCAGCCAAGCTGAAGAG	CCGGGCACTTTCCTTAGGT
*GNAO1*	CCAAATACTACCTGGACAGCCT	TGGGTTTCTACGATGCCAGT
*GNB1*	TTGTGATGCTTCAGCCAAAC	TTTGGAAAGAAGCAAATGGC
*GNB2*	CCACACTGGGTACCTGTCG	GTCTGCTGGCCTGTCTCAAT
*GPI*	CCAAGTCCAGGGGCGTG	CTTGTTGACCTCTGGCATCACA [50]
*GPX1*	AGTTTGGGCATCAGGAGAAC	GCACTTCTCGAAGAGCATGA
*GPX3*	AAGTCGAAGATGGACTGCCA	CCAGCATACTGCTTGAAGGG
*GPX7*	AACAGGAGCCTGACAGCAAC	AGTCTGGGCCAGGTACTTGAA
*GSK3a*	GGTTCAAGAACCGAGAGCTG	TCGTCTTTCTTCTCGCCACT
*GSK3b*	CGGGATATTAAACCGCAGAA	CGAAACATTGGGTTCTCCTC
*HADH2/HSD17B10*	CTGTCAACTGTGCAGGCATC	GCCCATGAGATTCACATCAAG
*HDAC1*	AGCCGGTCATGTCCAAAGTA	TTGGCGTGTCCTTTGATAGTT
*HMGCR*	TCTTCCACGTGCTTGTGACT	CGTGCAAATCTGCTAGTGCT
*HPRT1*	TGACACTGGCAAAACAATGCA	GGTCCTTTTCACCAGCAAGCT [26]
*IDH1*	TTTGAAGAAGGTGGTGGTGTT	TCAGATACAAAGGCCAACCC
*IL1A*	GCAGTGAAATTTGACATGGGT	ATCTCCTTCAGCAGCACTGG
*INSR*	TATTGCCTCAAAGGGCTGAA	CTTTGGACAGGAGCAGCATT
*LPL*	GGGCATGTTGACATTTACCC	GCTGGTCCACATCTCCAAGT
*LRP1*	TGGACTACCAGGATGGGAAG	ATGTCCATGTTGTTGCTGGA
*LRP6*	CATGGGCCTAAAGGCTACAA	TTCAAAGCCAATAGGGCAAG
*MAP2*	AATACAGCCCACCTCAGCAG	GGAGGAAGGTCTTGGGAGAG
*MAPT*	AGCCAAGACATCCACACGTT	AGGGTTGGATCAGAGGGTCT
*MMP2*	ACGACCGCGACAAGAAGTAT	ATTTGTTGCCCAGGAAAGTG
*MPRIP*	ATCTCAGCCATCGAAGCCAT	TGGCTCTTCTCCAGCTCCC
*NQO1*	AAAGGACCCTTCCGGAGTAA	CTCTGAATTGGCCAGAGAATG
*NUDT15*	CCAACTCCCTGGAGGTCA	AGCTGCTTCTTCCCAGGTTT
*PARK7*	TGGTGGTTCTACCAGGAGGT	GTAGGACCTGCACAGATGGC
*PKP4*	ACAGCATCTGGGACCTTCAC	GGAACTCCGTAAGCCTGTCA
*PLAT*	GAGTGCACCAACTGGAACAG	TAGCACCAGGGCTTTGAGTC
*PLAU*	ACGACATTGCCTTGCTGAAG	GGCAGGCAGATGGTCTGTAT
*POLR2F*	CCCGAAAGATCCCCATCAT	CACCCCCCAGTCTTCATAGC
*PPP1R15B*	AGGTAGTCGGCTTCCAGACA	AGGCCTTCCGTAGAAAGAGG
*PRDX1*	CAACTGCCAAGTGATTGGTG	CCAAAGGAATGTTCATGGGT
*PRDX2*	CACCTGGCTTGGATCAACA	GCCGTAATCCTCAGACAAGC
*PRDX6*	TTGTGAAGAGCCCACAGAAA	AACAAACACCACACGAGCTG
*PRKAA1*	TGCACACATGAATGCAAAGA	GGCCTGCATACAATCTTCCT
*PRKAA2*	CCAAATTATGCAGCACCTGA	CATGCTCATCATCAAATGGG
*PRKCA*	ACTTCATGGGATCCCTTTCC	TCCATGTTTCCTTCCTCGTC
*PRKCD*	AGGATGTGGTCCTGATCGAC	AACAGGTGGTCCTTGGTCTG
*PRKCE*	AGCTTTGGCAAGGTCATGTT	CAGTCCACGTCATCATCCTG
*PRKCG*	CAGGAGGAGGGCGAGTATTA	ACCCGCTCATACAATTCCAG
*PRKCQ*	CGAGAAACCATGTTCCACAA	GGTCCCACAGTGTTCACAGA
*PRKCZ*	ACAGACTACGGCATGTGCAA	ACGCTGAACCCGTACTCCT
*PRNP*	AACAAGCCGAGTAAGCCAAA	AAATGTATGATGGGCCTGCT
*PSENEN*	CATCTTCTGGTTCTTCCGAGAG	AGAAGAGGAAGCCCACAGC
*PSMB4*	CTTGGTGTAGCCTATGAAGCCC	CCAGAACTTCTCGCAGCAGAG [50]
*RN18S1*	GGAGTATGGTTGCAAAGCTGA	ATCTGTCAATCCTGTCCGTGT [51]
*RPL13A*	CCTGGAGGAGAAGAGGAAAGAGA	TTGAGGACCTCTGTGTATTTGTCAA [26]
*SDHA*	TGGGAACAAGAGGGCATCTG	CCACCACTGCATCAAATTCATG [26]
*SH2D3C/CHAT*	CCAAGGAGATGCAGACCCTA	TGTGGAACCTTTCCAGCAG
*SNCA*	ACCAGTTGGGCAAGAATGAA	CCCTTCCTCAGAAGGCATTT
*SNCB*	CTGGGAGGAGCTGTGTTCTC	CCACTTCCTCTGGCTTCAGAT
*SNRPD3*	TGCCAGATGTCCAACATCACAGTC	ACATGGGTGCGTTCTTCAGCA
*SOD1*	CAGCAGGCTGTACCAGTGC	ACATTGCCCAAGTCTCCAAC
*TBP*	CGGAGAGTTCTGGGATTGT	GGTTCGTGGCTCTCTTATC
*TOP1*	TGAAAGTCCGGCAGAGAGCTG	GCCCACAGTGTCCGCTGTTT
*TXNIP*	TGTGAAGGTGATGAGATTTCCA	GCCATTGGCAAGGTAAGTGT
*TXNRD2*	GCATGACTGGAGGAAGATGG	AAACCGTGTGCTCGTCAAC
*UBC*	GTGTCTAAGTTTCCCCTTTTAAGG	TTGGGAATGCAACAACTTTATTG [52]
*UBQLN1*	GCAACCTAGAAAGCATCCCA	TGGATTACCACCAAACTGCTC
*UCP3*	ATTTCAGGCCAGCATACACC	GGCAAAGTTCCTTTCCACAG
*UQCRC1*	TGTCAGGAAGCTGTCTCGTG	TCTGGGCGAGGTCTAACAGT
*UQCRC2*	ATGGCTTTGATTGGACTTGG	TTCACCTCCACGGTAGTTGG
*XPA*	CAACCAGGACCTGTTATGGAA	TGCAGTTATCACAAGTTGGCA
*YWHAZ*	ACTTTTGGTACATTGTGGCTTCAA	CCGCCAGGACAAACCAGTAT [26]

## Supplementary Materials

Supplementary Materials can be found at https://www.mdpi.com/1422-0067/21/23/9015/s1.
