# Supplementary material for: Unique Role of Caffeine Compared to Other Methylxanthines (Theobromine, Theophylline, Pentoxifylline, Propentofylline) in Regulation of AD Relevant Genes in Neuroblastoma SH-SY5Y Wild Type Cells
Source: Int J Mol Sci. 2020 Nov 27;21(23):9015. doi: 10.3390/ijms21239015 (PMC7730563; doi:10.3390/ijms21239015)
Supplement: Supplementary file 1 [file ijms-21-09015-s001.pdf]

Supplementary information

Supplement S1. *Detailed overview of the genes that were selected for examination of methylxanthines-caused transcriptional changes and their classification in pathways.*

gene	gene name	reference
**oxidative stress**		
CAT	Catalase	- depletion of cellular hydrogen peroxide<br>- deficiency or malfunction of catalase can be linked to degenerative diseases [1]<br>- there is a broad agreement in the role of oxidative stress in the development of cardiovascular injuries [2]<br>- oxidative stress is reported as important pathophysiological change in COPD [3]
GPX1	Glutathione peroxidase 1	- detoxification of H_2O_2<br>- GPX shows an age related reduced activity and are decreased in plasma samples of AD patients [4,5]<br>- modulation of GPx-3 activity is associated with altered risk of cardio-renal disease [6]<br>- a study reported a reduced activity of GPx in COPD cases compared to healthy controls [7]
GPX3	Glutathione peroxidase 3	
GPX7	Glutathione peroxidase 7	
NQO1	NAD(P)H dehydrogenase, quinone 1	- polymorphism in NQO1 as a risk factor for sporadic AD [8,9]<br>- gender-dependent (women) association with coronary artery disease and NQO1 polymorphism was found [10]
NUDT15	Nudix type motif 15	- Methylation levels of NUDT15 were altered in mild cognitive impairment (MCI) patients [11]
PARK7	Parkinsonism associated deglycase	- up-regulated DJ-1 expression related to oxidative stress and AD [12]<br>- PARK7 protein found to be regulating vascular contractility and blood pressure, thus being a risk factor for cardiovascular diseases [13]<br>- Protein levels of PARK7 were decreased in the lungs of COPD patients [14]
PPP1R15B	protein phosphatase 1, regulatory subunit 15B	- altered expression of PPP1R15B in murine cardiac tissue after intestinal ischemia-reperfusion [15]<br>- inhibitors of PPP1R15B were shown to be beneficial in a model of neurodegenerative disease [16]
PRDX1	Peroxiredoxin 1	- a correlation of peroxiredoxin isoforms has been shown for traditional cardiovascular risk factors [17]<br>- significant up-regulation of PRDX1 gene could be seen in COPD patients versus healthy smokers [18]
PRDX2	Peroxiredoxin 2	
PRDX6	Peroxiredoxin 6	
SOD1	Superoxide dismutase 1	- SOD1 deficiency drives Aβ loss [19]<br>- a prospective study with 2799 subjects showed an association with CV deaths in the general population with a variation in the SOD1 gene [20]<br>- SOD1 and SOD3 show associations between polymorphisms and increased risk for COPD [21]
TXNIP	Thioredoxin interacting Protein	- overexpression of TXNIP in hippocampus of AD mice model [22]<br>- TXNIP binds to thioredoxin (TRX) causing inhibition of its antioxidant activity [22]<br>- TXNIP function as a TRX inhibitor leads to pathological effects in diabetes and cardiovascular disease [23]<br>- a lower expression of TXNIP was revealed in COPD subjects than in smokers, the gene could distinguish smokers from COPD subjects [24]
TXNRD2	Thioredoxin reductase 2	- KO of heart specific mt TXNRD2 in mice showed an abnormal heart development which is lethal to mice embryos; inactivation led to dilated cardiomyopathy [25]<br>- study showed a negative correlation between mRNA levels of TXNRD2-3 and age in lung [26]
UCP3	uncoupling	- 1057 European adolescents from the HELENA study have shown UCP3 polymorphisms

	protein 3	which are associated with CVD risk factors/risk score [27]<br>- impairs myocardial fatty acid oxidation (mice model) [28]<br>- UCP3 protein levels were decreased in COPD patients compared to a healthy age matched control, the study could show a significant correlation between UCP3 content and FEV1 [29]
UQCRC1	ubiquinol-cytochrome c reductase core protein I	- overexpression of UQCRC1 has shown a protective effect in H9c2 cardiac cells against simulated ischemia/reperfusion via zinc binding [30]<br>- was found to be significantly differently expressed in GO enrichment analysis in patients during AECOPD [31]
UQCRC2	ubiquinol-cytochrome c reductase core protein II	- significantly increased UQCRC2 protein levels were found in patients with CAD compared to non-CAD patients [32]<br>- known interaction between NLRX1 via binding at UQCRC2 in respiratory chain complex III regulating ROS production in mitochondria - NLRX1 expression is decreased in patients with COPD [33]
CDK5	Cyclin-dependent kinase 5	- substrates of Cdk5 are APP and tau; deregulation of Cdk5 leads to an array of pathological events in AD [34]<br>- activity and expression of Cdk5 is stimulated by oxidative stress [35]<br>- in the context of cardiovascular diseases targeted inhibition of Cdk5 is discussed as potential therapeutic target [36]
DUOX1	Dual oxidase 1	- DUOX1 is an H_2O_2 producing oxidative stress related gene [37]<br>- NADPH oxidases play a role in neurodegenerative diseases [38]<br>- DUOX-mediated H_20_2 is linked to different features of allergic asthma and oxidative stress, that is an important pathogenesis of cardiovascular diseases [39]
IDH1	Isocitrate dehydrogenase 1 ($NADP^+$)	- even the oxidative stress but also the nitrosative stress are dependent on the deletion of IDP1 [40]<br>- it was observed, that IDH1 could be linked to alterations in the energy metabolism that is a late pathomechanism for Alzheimer's Disease [41]
ERCC2	ERCC excision repair 2 TFIIH core complex helicase subunit	- higher expression of excision repair-cross-complementing (ERCC) proteins in brains of patients with AD [42]<br>- a role for DNA repair proteins is described in atherosclerosis and cardiovascular diseases [43]<br>- ERCC proteins participates in the repair of oxidative stress induced lesions [44]
ERCC6	ERCC excision repair 6, chromatin remodeling factor	
XPA	xeroderma pigmentosum, complementation group A	- molecular misreading in AD [45]<br>- a strong correlation between XPA expression and fibrous plaques was observed in NSTEMI patients [46]<br>- genes which demonstrated a high module membership and gene significance for clinical indices of COPD severity included XPA (& ERCC5) [47]

**lipid & energy metabolism**		
ABCA1	ATP-binding cassette, subfamily A, member 1	- ABCA1 has a regulatory role in brain amyloidogenesis and amyloid clearance [48]<br>- an ABCA1 gene variation was reported to be associated with the risk of a heart disease during pravastatin treatment [49]<br>- ABCA1 is discussed as therapeutic target for therapy of cardiovascular diseases (cholesterol is major cardiovascular disease risk factor) [50]<br>- as reviewed in [51] ATP-binding cassette transporters appear to have a crucial and protective role in respiratory diseases
ACAT1	Acetyl-CoA acetyltransferase 1	- cholesteryl ester levels were correlated with Aβ generation and blocking ACAT activity diminished Aβ generation [52,53]<br>- polymorphism in the ACAT1-gene is linked with a higher risk of coronary artery disease (CAD) [54]<br>- expression of ACAT1 seems to be induced by oxidative stress [55]
APOA1	Apolipoprotein A-I	- the HDL-cholesterol transporter ApoA1 inhibits amyloid-beta aggregation [56]<br>- Abeta production is influenced by cholesterol levels [57]<br>- The ratio of ApoB and ApoA1 is suggested as marker for atherosclerotic risk in prevention of coronary artery disease [58]<br>- ApoA1 is suggested to be beneficial in the prevention and the treatment of lung diseases [59]<br>- overexpression of ApoA1 suppressed the generation of ROS in lungs of cigarette smoke exposed mice [60]
HMGCR	3-hydroxy-3-methylglutaryl-CoA reductase	- AD risk is associated with HMGCR gene [61]<br>- variants of HMGCR and PCSK9 have similar effects on the risk of cardiovascular events and diabetes in correlation to the LDL cholesterol level [62]
APOE	Apolipoprotein E	- ApoE is co-deposited in senile plaques in AD brains [63]<br>- a primarily protective role in lung biology and respiratory disease is observed for ApoE in different studies [64]<br>- ApoE and its isoforms play a role in the pathomechanism of cardiovascular diseases [65]<br>- deficiency of ApoE promotes increased oxidative stress [66]
HADH2	3-hydroxyacyl-CoA dehydrogenase type II	- Hadh2 is interacting with the amyloid-beta peptide [67]
LRP6	Low density lipoprotein receptor-related protein 6	- Alzheimer´s Disease is associated with a single-nucleotide polymorphism in exon 18 of LRP6 [68]<br>- association between a mutation in LRP6 (R611C) and early coronary artery disease was observed [68]
LPL	Lipoprotein lipase	- LPL accumulation in Abeta plaques; polymorphisms of lipoprotein lipase associated with AD risk [69-71]<br>- LPL stimulation could probably lead to lower plasma lipids that could have a positive impact of cardiovascular outcomes [72]
INSR	Insulin receptor	- significant upregulation of INSR expression in the hippocampus and the entorhinal cortex in AD [73]<br>- defective INSR leads to an acceleration of atherosclerosis which is a cause of cardiovascular diseases [74]<br>- INSR is down-regulated in diseases like Asthma and COPD [75]
PRKAA1	Protein kinase, AMP-activated, alpha 1 catalytic subunit	- AMPK can phosphorylate and directly activate PGC-1alpha and can also phosphorylate substrates including Tau protein, leading to tau hyperphosphorylation [76]<br>- PRKAA1 deletion accelerated atherosclerotic lesions in hyperlipidemic mice [77]<br>- encodes AMPKa1 which is involved in pathological changes in vessels of pulmonary hypertension patients [78]

PRKAA2	Protein kinase, AMP-activated, alpha 2 catalytic subunit	- altered expression of *PRKAA2* in APP/PS1 mice [79]
ERN1	Endoplasmatic reticulum to nucleus signaling 1	- polymorphism in the *ERN1* promoter are discussed as risk factor for the development of AD [80]<br>- Evidence arise that ERN1 plays an important role in cardiovascular disease [81]
CLU	Clusterin	- CLU is a cellular biosensor of oxidative load alterations and is linked to different pathological conditions like atherosclerosis [82]<br>- in regard to AD clusterin was identified as Aβ toxicity mediator [83]
AASS	Aminoadipate-semialdehyde synthase	- AD is associated with protein carbonylation [84]<br>- hyperexpression of this gene in cardiac tissue is discussed as protection against the harmful action of ROS [15]

signal transduction & gene expression		
PRKCA	Protein kinase C, alpha	- direct activation of the alpha secretase mediated cleavage of APP;<br>gain-of-function-mutations in PKCalpha may promote synaptic defects [85,86]<br>- Protein kinase C activation has shown a link to cardiovascular disease states as reviewed in [87]
PRKCD	Protein kinase C, delta	- PKCd-/- mice showed an attenuation of pulmonary fibrosis via inhibition of the NF-KB [88]
PRKCE	Protein kinase C, epsilon	- direct activation of the alpha secretase mediated cleavage of APP [85]<br>- PRKCE showed a strong association with forced one second expiratory volume and the FEV1/FVC ratio [89]
PRKCG	Protein kinase C, gamma	- decreased PKCG level in AD fibroblasts [90]
PRKCQ	Protein kinase C, theta	- PRKCQ was reported to influence the permeability of the blood-brain barrier via tight junction protein disruption [91]<br>- PRKCQ is an important intermediate in signaling pathways for a robust lung inflammatory response [92]
PRKCZ	Protein kinase C, zeta	- PRKCZ may contribute to the development of cardiomyopathy [93]<br>- Patients with AECOPD showed a dynamic gene expression in an up-up pattern [94]
ALS2	alsin Rho guanine nucleotide exchange factor	- Alsin has neuroprotective activity against ALS-related insults [95]<br>- ALS2-KO neurons are reported to be predisposed to oxidative stress [96]
GNAO1	G protein subunit alpha o1	- It is discussed that PS1 can interact and regulate G protein activity [97]<br>- Aβ can influence function of G-proteins and its receptors directly or indirectly [98]<br>- G proteins are related to cardiovascular diseases [99]<br>- first signal transduction cascade in response to oxidative stress includes G proteins
GNB1	Guanine nucleotide binding protein (G protein) beta polypeptide 1	
GNB2	Guanine nucleotide binding protein (G protein) beta polypeptide 2	
EP300	E1A (adenovirus early region 1A) binding protein p300	- Ep300 is dysregulated in AD brains compared to healthy controls [100]<br>- alterations of histone acetylation on PS1- and BACE1 promoters were described to be mediated by EP300 [101]<br>- EP300 was linked to the development of recurrent cardiovascular events [102]
HDAC1	Histone deacetylase 1	- in AD patients the level of HDAC1 is significantly attenuated [103]<br>- the HDAC activity reflects the severity of COPD [104]<br>- HDACs are regulators that are playing a role in certain cellular processes e.g. apoptosis, fibrosis, oxidative stress and inflammation [105]

Aβ- and tau pathology & inflammation		
CDKL1	Cyclin-dependent kinase-like 1	- Cdkl1 is a kinase involved in the pathologic phosphorylation of tau, a neuropathological feature of AD [106]
GAP43	Growth associated protein 43	- increased GAP43 mRNA and protein level were reported in AD brains [107,108]
GSK3a	Glycogen synthase kinase 3 alpha	- GSK3 is a key kinase contributing to abnormal tau-phosphorylation causing neurofibrillary tangles in AD [109,110]<br>- GSK3 is discussed as potential therapeutic target for myocardial diseases [111]<br>- GSK3b mediates ROS-induced glucocorticoid unresponsiveness in COPD [112]
GSK3b	Glycogen synthase kinase 3 beta	
APBA1	APP binding, family A, member 1	- APBA proteins fine-tune APP trafficking and processing [113,114]<br>- regulation of APP processing [115]<br>- interaction of APP with APP-BP1 is required for activation of DNA synthesis and apoptosis in neurons [116]<br>- APP and its processing products are reported to be present in artherosclerotic plaques, which are part of the pathomechanism of cardiovascular diseases [117,118]<br>- circulating Aβ40 levels were predictive of cardiovascular mortality in patients with coronary heart disease (CHD) as reported in a retrospective cohort study [119]<br>- serum levels of Aβ40, Aβ42 and total Aβ are significantly increased in patients with chronic obstructive pulmonary disease compared to healthy controls [120]
APBA3	APP binding, family A, member 3	
APBB1	APP binding, family B, member 1	
APBB2	APP binding, family B, member 2	
APPBP1/NAE1	APP binding protein 1	
UBQLN1	ubiquitilin 1	- UBQLN1 expression was decreased in human temporal cortex in relation to the early stages of AD related neurofibrillary pathology [121]<br>- Ubqln1-CKO mice showed an increase in myocardial ubiquitinated proteins, leading to late-onset cardiomyopathy and reduced life span [122]<br>- bioinformatical analysis showed UBQLN1 as an DEG in cases with severe COPD [123]
APH1a	aph1 homolog A, gamma secretase subunit	- encodes a component of the gamma secretase complex [124-126]<br>- the pathogenesis of different lung diseases has been linked to the regulation of the Notch signaling, a substrate of γ-secretase [127]<br>- Notch is suggested to be involved in the aetiology of different cardiovascular diseases and discussed as therapeutic target [128]<br>- the γ-secretase is discussed to mediate the oxidative stress-induced increase of the beta-secretase enzyme [129]
APH1b	aph1 homolog B, gamma secretase subunit	
CASP3	caspase 3	in AD cases there is a high degree of co-localization of activated caspases and neurofibrillary tangles / senile plaques (see also CASP4)
CTSB	cathepsin B	- Cathepsin was described to play a role in APP metabolism [130]<br>- in AD impairments in the cathepsin family have been observed [131]<br>- Plasma cathepsins are potential biomarkers for cardiovascular disease in humans [132]<br>- Cathepsin L is discussed to degrade an alternative APP c-terminal fragment [133]
CTSL	cathepsin L	
PSENEN	presenilin enhancer, gamma secretase subunit	- encodes a component of the gamma secretase complex [126] (see also APH1a and APH1b)
A2M	alpha-2-macroglobulin	- A2m is associated with cerebrospinal fluid (CSF) markers of neuronal injury in preclinical AD [134]<br>- A2M gene polymorphism is associated with Parkinson´s disease [135]<br>- up-regulated in a model of dyssynchrony heart failure (DHF) [136]<br>- up-regulated protein expression in ST-elevation myocardial infarction [137]
ECE1	endothelin converting enzyme 1	- Aβ-degrading enzymes [138] (see also "APP binding and transport" section)
ECE2	endothelin converting enzyme 2	
LRP1	low density lipoprotein receptor-related protein 1	- LRP is associated with the transport, the production and the clearance of Abeta [139]<br>- Inactivation of LRP1 leads to a higher risk of atherosclerosis and could result in aneurysm formation [140]
MMP2	matrix metallo-peptidase 2	- Aβ-Degradation [141]<br>- functional polymorphisms of MMPs were related to arterial stiffness and CAD [142]

		- MMP members are up-regulated during acute or chronic phase of lung diseases [143]
PKP4	plakophilin 4	- interaction with presenilin 1 [144]
PRNP	prion protein	- Significantly altered levels of PRNP mRNA levels in ventricular autopsy samples from children and young adults which were exposed to high concentrations of air pollutants indicating increased myocardial inflammation [145]<br>- Significant association with COPD was found only in smokers [146]
SNCA	synuclein, alpha	- up-regulated expression of SNCA in AD subjects than in control subjects [147]<br>- link between alpha-synuclein levels in the blood and insulin resistance in SNCA knock-out mice; SNCA association to diabetes indirectly links it to CVD as diabetes is a risk factor [148]
SNCB	synuclein, beta	- levels of beta-synuclein were decreased in AD [149]
CASP4	Caspase 4	- in AD cases there is a high degree of co-localization of activated caspases and neurofibrillary tangles / senile plaques [150,151]<br>- anti-apoptotic approaches seem to be an effective therapeutic strategy for cardiovascular diseases [152]<br>- in the pathogenesis of COPD apoptosis of structural lung cells might possibly be an important event [153]
IL1A	Interleukin 1, alpha	- Altered expression is observed in AD [154,155]<br>- IL-1 cytokines and the inflammasome are linked to the pathogenesis of cardiovascular diseases [156]<br>- IL-1 alpha is secreted via alveolar macrophages and leads to subsequent lung inflammation [157]
SH2D3C/CHAT	SH2 domain containing 3C	- SH2D3C is reported to be overexpressed in Aß-enriched fractions [158]<br>- expression of SH2D3C was found to correlate negatively with smoking, a risk factor for COPD, in a transcriptomic study in lymphocytes [159]

neuronal genes		
MAP2	Microtubule-associated protein 2	- Map2 is negative correlated to the pathomechanism of AD [160]<br>- As a part of the MAPK-Cascade Map2 is involved in pulmonary cell adhesion and cell motility [161]
MAPT	Microtubule-associated protein tau	- hyperphosphorylated tau → neurofibrillary tangle [162,163]<br>- top gene identified in COPD patients vs control group in a sherlock analysis [164]
PLAU	Plasminogen activator, urokinase	- Polymorphism in PLAU gene could increase the risk of AD [165]<br>- Gene variations of the PLAU gene were significantly associated with an increased risk of MI [166]<br>- via activation of TGF-beta1 inhibition of SERPINE2, PLAU could be linked to COPD [167]
PLAT	Plasminogen activator, tissue	- t-PA is associated with Abeta degradation [168]<br>- t-PA protein level were significantly lower for healthy control patients than CAD patients [169]
ACHE	Acetylcholinesterase	- decreased AChE activity in AD brains [170,171]<br>- Treatment with acetylcholinesterase inhibitors was linked to a reduced risk of acute coronary syndrome in patients with dementia [172]<br>- Alterations in AChE activities can be associated with development and severity of COPD [173]<br>- AChE was reported to regulate lipid metabolism in the brain via transcriptional influence on lipid-related genes [174]
BDNF	Brain-derived neurotrophic factor	- declined BDNF level in AD leads to poorly differentiated neurons, synapse loss and cognitive dysfunction [175-177]<br>- A direct interplay of oxidative stress indicators and the levels of BNDF is suggested in the brain [178]<br>- BDNF is indicated as important biomarker associated with parameters of COPD severity [179]<br>- reduced plasma BDNF concentration is suggested to be linked to coronary heart disease pathogenesis [180]

Supplement S2. Selection of stable housekeeping genes. *The table listed the selected housekeeping genes and their expression stability calculated by NormFinder in SH-SY5Y-, HEPG2- and Calu-3 cells treated with different xanthine derivatives. The figure shows the expression levels of the selected genes for normalization given as quantitative real-time PCR cycle treshold (Cq) values in Calu-3(A) and HEPG2 (C) cells. Expression stability values for 19 analyzed genes calculated by NormFinder algorithm. Housekeeping genes are ranked from the least to the most stable one from the left to right and the five most stable genes selected for normalization in Calu-3 (B) and HEPG2 (D) cells are highlighted in a box.*

gene	gene name	SH-SY5Y	HEPG2	Calu-3
ACTB	Actin Beta	0.165	0.133	0.159
ATP5B	ATP synthase F1 subunit beta, mitochondrial	0.149	0.117	0.188
B2M	Beta-2-Microglobulin	0.218	0.207	0.359
C1ORF43	Chromosome 1 open reading frame 43	0.407	0.137	0.237
EIF4A2	Eukaryotic translation initiation factor 4A2	0.177	0.103	0.242
EMC7	ER membrane protein complex subunit 7	0.131	0.122	0.284
GAPDH	Glyceraldehyde-3-phosphate-dehydrogenase	0.106	0.179	0.233
GPI	Glucose-6-phospate isomerase	0.120	0.154	0.273
HPRT1	Hypoxanthine phosphoribosyltransferase 1	0.168	0.133	0.222
MPRIP	Myosine phosphatase Rho interacting protein	0.149	0.466	0.445
POLR2F	RNA polymerase II,I and III subunit F	0.139	0.146	0.297
PSMB4	Proteasome 20F subunit beta 4	0.130	0.116	0.169
RN18S1	18S ribosomal RNA	0.126	0.163	0.259
RPL13A	Ribosomal protein L13a	0.178	0.163	0.295
SDHA	Succinate dehydrogenase complex flavoprotein subunit A	0.136	0.121	0.218
SNRPD3	Small nuclear ribonucleoprotein D3 polypeptide	0.127	0.106	0.318
TBP	TATA-box binding protein	0.132	0.143	0.246
TOP1	DNA topoisomerase 1	0.109	0.133	0.326
YWHAZ	Tyrosine 3-monooxygenase/tryptophan 5-monooxygenase activation protein zeta	0.107	0.146	0.130

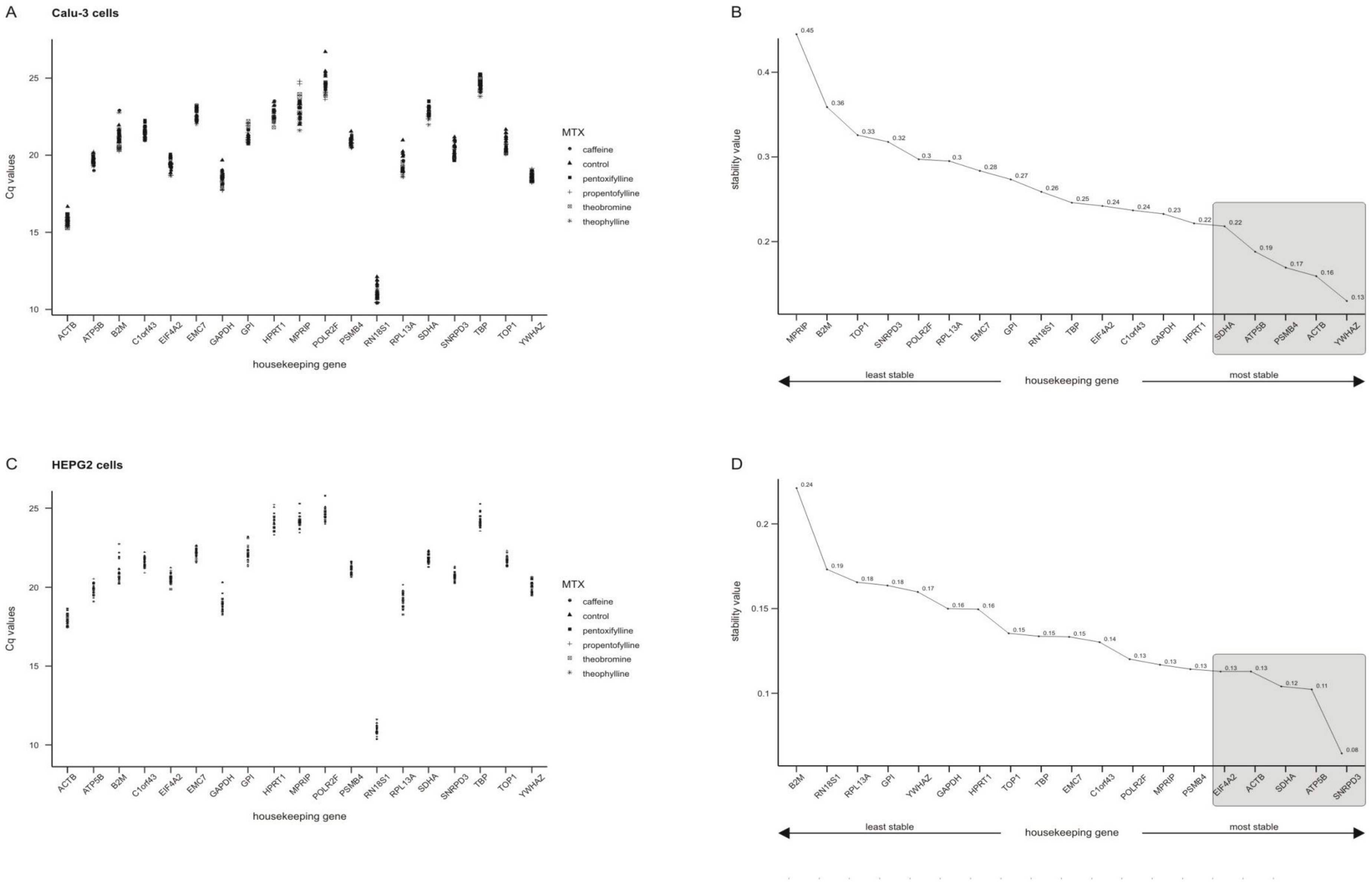
A
Calu-3 cells
B
C
HEPG2 cells
D
Cq values
housekeeping gene
stability value
MTX
caffeine
control
pentoxifylline
propentofylline
theobromine
theophylline
ACTB ATP5B B2M C1orf43 EIF4A2 EMC7 GAPDH GPI HPRT1 MPRIP POLR2F PSMB4 RN18S1 RPL13A SDHA SNRPD3 TBP TOP1 YWHAZ
MPRIP B2M TOP1 SNRPD3 POLR2F RPL13A EMC7 GPI RN18S1 TBP EIF4A2 C1orf43 GAPDH HPRT1 SDHA ATP5B PSMB4 ACTB YWHAZ
0.45 0.36 0.33 0.32 0.3 0.3 0.28 0.27 0.26 0.25 0.24 0.24 0.23 0.22 0.22 0.19 0.17 0.16 0.13
B2M RN18S1 RPL13A GPI YWHAZ GAPDH HPRT1 TOP1 TBP EMC7 C1orf43 POLR2F MPRIP PSMB4 EIF4A2 ACTB SDHA ATP5B SNRPD3
0.24 0.19 0.18 0.18 0.17 0.16 0.16 0.15 0.15 0.15 0.14 0.13 0.13 0.13 0.13 0.13 0.12 0.11 0.08
least stable
most stable

Supplement S3. Rawdata after incubation in SH-SY5Y cells. *Detailed overview of transcriptional changes after treatment with caffeine, theobromine, theophylline, pentoxifylline, and propentofylline in SH-SY5Y cells compared to treatment with water as solvent control. Included are the mean expression changes ± standard error of the mean (SEM) after normalization using the five most stable housekeeping genes (RN18S1, GPI, TOP1, YWHAZ, and GAPDH). P values for ANOVA analysis, false discovery rate adjusted p values ("ANOVA adj.") and p values for Dunnett´s Test (each methylxanthine in comparison to the solvent control) are shown.*

gene	mean expression changes ± standard error of the mean (SEM) [%]						p values						
	control	caffeine	theobromine	theophylline	pentoxifylline	propento-fylline	ANOVA	ANOVA adj.	Ctrl vs. C	Ctrl vs. TB	Ctrl vs. TP	Ctrl vs. P	Ctrl vs. PF
CAT	*100 ± 6.96*	*130.07 ± 13.76*	*105.52 ± 16.49*	*97.07 ± 19.6*	*93.87 ± 26.82*	*100.54 ± 26.68*	*0.8205*	*0.8407*					
GPX1	*100 ± 4.64*	*114.58 ± 4.78*	*80.41 ± 2.13*	*83.78 ± 10.11*	*108.88 ± 6.99*	*77.58 ± 8.35*	*0.0012*	*0.0038*					
GPX3	*100 ± 18.95*	*153.5 ± 10.69*	*86.97 ± 5.54*	*97.38 ± 21.94*	*118.42 ± 28.29*	*98.46 ± 19.69*	*0.2976*	*0.3828*					
GPX7	*100 ± 10.69*	*119.98 ± 2.38*	*92.73 ± 8.12*	*100.99 ± 6.86*	*96.56 ± 11.83*	*83.71 ± 11.75*	*0.1421*	*0.2184*					
NQO1	*100 ± 8.99*	*157.56 ± 26.24*	*94.45 ± 5.29*	*117.05 ± 3.69*	*97.76 ± 25.43*	*105.93 ± 7.77*	*0.2962*	*0.3828*					
NUDT15	*100 ± 8.72*	*118.38 ± 4.79*	*104.7 ± 7.95*	*103 ± 2.35*	*95.56 ± 5.92*	*80.58 ± 8.71*	*0.0227*	*0.0449*					
PARK7	*100 ± 5.85*	*132 ± 2.39*	*93.58 ± 5.54*	*82.79 ± 10.47*	*103.2 ± 9.4*	*67.67 ± 4.53*	*0.0000*	*0.0003*	*0.0104*	*0.2507*	*0.9961*	*0.9277*	*0.0098*
PPP1R15B	*100 ± 5*	*115.76 ± 14.3*	*97.2 ± 3.55*	*94.73 ± 1.48*	*105.72 ± 5.37*	*90.33 ± 5.62*	*0.2997*	*0.3828*					
PRDX1	*100 ± 7.25*	*141.78 ± 5.73*	*168.39 ± 9.27*	*114.23 ± 16.05*	*163.31 ± 10.56*	*169.88 ± 6.96*	*0.0063*	*0.0160*	*0.1606*	*0.9168*	*0.0182*	*0.0104*	*0.0088*
PRDX2	*100 ± 9.97*	*96.14 ± 6.65*	*107.91 ± 3.78*	*97.04 ± 3.36*	*120.79 ± 7.02*	*92.91 ± 3.49*	*0.0645*	*0.1071*					
PRDX6	*100 ± 14.17*	*124.25 ± 7.12*	*169.35 ± 7.04*	*137.95 ± 16.42*	*202.09 ± 6.14*	*185.26 ± 5.22*	*0.0005*	*0.0021*	*0.6276*	*0.2404*	*0.0003*	*0.0109*	*0.0020*
SOD1	*100 ± 5.97*	*96.13 ± 9.41*	*69.06 ± 8.37*	*76.85 ± 10.4*	*82.38 ± 5.58*	*69.75 ± 9.84*	*0.0181*	*0.0396*	*0.9931*	*0.1044*	*0.2814*	*0.0208*	*0.0241*
TXNIP	*100 ± 8.23*	*61.45 ± 31.93*	*77 ± 7.88*	*47 ± 9.56*	*109.24 ± 15.95*	*60.54 ± 11.75*	*0.0113*	*0.0253*	*0.1284*	*0.0238*	*0.9733*	*0.5383*	*0.1162*
TXNRD2	*100 ± 2.18*	*125.01 ± 2.79*	*80.3 ± 5.82*	*92.42 ± 4.97*	*112.07 ± 8.7*	*71.7 ± 4.64*	*0.0000*	*0.0001*	*0.0142*	*0.7623*	*0.3766*	*0.0626*	*0.0056*
UCP3	*100 ± 8.65*	*95.62 ± 18.31*	*106.26 ± 3.89*	*80.02 ± 4.18*	*110.46 ± 4.13*	*97.3 ± 10.38*	*0.3260*	*0.4042*					
UQCRC1	*100 ± 16.98*	*116.92 ± 10.24*	*113.55 ± 33.19*	*82.45 ± 31.22*	*125.7 ± 36.45*	*85.6 ± 28.95*	*0.8721*	*0.8721*					
UQCRC2	*100 ± 13.14*	*92.99 ± 5.65*	*94.25 ± 7.65*	*91.12 ± 7.29*	*95.81 ± 13.58*	*74.36 ± 13.36*	*0.5394*	*0.6133*					
CDK5	*100 ± 10.36*	*134.03 ± 5.4*	*81.63 ± 26.92*	*87.44 ± 3.1*	*115.11 ± 2.01*	*99.47 ± 9.55*	*0.0410*	*0.0708*					
DUOX1	*100 ± 9.67*	*70.95 ± 33.5*	*95.67 ± 8.77*	*105.04 ± 12.93*	*99.89 ± 26.56*	*72.87 ± 25.15*	*0.6432*	*0.6933*					

IDH1	*100 ± 12.75*	*103.92 ± 15.89*	*132.82 ± 6.52*	*84.64 ± 11*	*126.44 ± 13.45*	*147.64 ± 4.66*	*0.0203*	*0.0422*	*0.9995*	*0.8505*	*0.4479*	*0.2614*	*0.0563*
ERCC2	*100 ± 9.65*	*159.67 ± 5.94*	*69.13 ± 5.56*	*56.64 ± 7.42*	*73.4 ± 9.64*	*51.86 ± 11.07*	*0.0000*	*0.0000*	*0.0000*	*0.0017*	*0.0606*	*0.0252*	*0.0007*
ERCC6	*100 ± 19.21*	*122.49 ± 16.47*	*42.71 ± 19.37*	*55.13 ± 19.68*	*54.24 ± 28.28*	*46.77 ± 18.25*	*0.0043*	*0.0116*	*0.7143*	*0.1517*	*0.1403*	*0.0477*	*0.0705*
XPA	*100 ± 13.76*	*99.95 ± 15.86*	*81.8 ± 10.57*	*84.09 ± 8.27*	*63.22 ± 19.1*	*77.36 ± 14.78*	*0.2664*	*0.3625*					
ABCA1	*100 ± 13.09*	*66.3 ± 37.1*	*44.78 ± 7.5*	*62.84 ± 11.71*	*115.31 ± 4.43*	*35.26 ± 7.86*	*0.0011*	*0.0038*	*0.2175*	*0.1531*	*0.8366*	*0.0189*	*0.0058*
ACAT1	*100 ± 4.12*	*85.53 ± 10.67*	*90.99 ± 6.85*	*96.19 ± 5.14*	*95.36 ± 10.32*	*79.74 ± 7.85*	*0.3787*	*0.4491*					
APOA1	*100 ± 13.82*	*71.75 ± 23.65*	*237.71 ± 12.65*	*226.95 ± 19.76*	*127.97 ± 12.98*	*209.87 ± 9.7*	*0.0006*	*0.0023*	*0.9022*	*0.0122*	*0.9057*	*0.0066*	*0.0320*
HMGCR	*100 ± 13.95*	*99.64 ± 12.39*	*111.18 ± 12.78*	*82.69 ± 9.35*	*102.47 ± 10.09*	*117.27 ± 4.16*	*0.3774*	*0.4491*					
APOE	*100 ± 9.26*	*130.89 ± 11.4*	*119.13 ± 10.18*	*86.62 ± 17.77*	*99.9 ± 11.28*	*125.5 ± 13.12*	*0.1954*	*0.2845*					
HADH2	*100 ± 6.85*	*155.45 ± 9.14*	*80.25 ± 5.49*	*72.37 ± 6.69*	*96.62 ± 5.43*	*67.01 ± 2.85*	*0.0000*	*0.0000*	*0.0002*	*0.0608*	*0.9972*	*0.2435*	*0.0210*
LRP6	*100 ± 16.73*	*146.35 ± 10.85*	*99.38 ± 12.02*	*95.19 ± 11.22*	*134.5 ± 5.27*	*91.43 ± 7.56*	*0.0188*	*0.0401*					
LPL	*100 ± 11.06*	*216.3 ± 9.25*	*219.24 ± 13.59*	*190.86 ± 7.38*	*132.87 ± 22.68*	*125.92 ± 22.52*	*0.0064*	*0.0160*	*0.0107*	*0.0532*	*0.7821*	*0.0090*	*0.8963*
INSR	*100 ± 27.26*	*126.57 ± 5.41*	*105.16 ± 22.84*	*112.63 ± 23.63*	*144.84 ± 33.68*	*132.17 ± 13.63*	*0.8611*	*0.8716*					
PRKAA1	*100 ± 16.41*	*111.83 ± 6.39*	*113.16 ± 8.29*	*92.5 ± 17.53*	*119.56 ± 16.86*	*122.71 ± 9.37*	*0.6532*	*0.6950*					
PRKAA2	*100 ± 25.87*	*113.39 ± 12.07*	*119.15 ± 11.84*	*146.81 ± 17.85*	*138.09 ± 34.82*	*95.27 ± 18.05*	*0.7167*	*0.7530*					
ERN1	*100 ± 11.13*	*183.32 ± 24.06*	*52.23 ± 13.12*	*47.46 ± 14.63*	*61.26 ± 7.84*	*55.8 ± 17.34*	*0.0007*	*0.0026*	*0.0292*	*0.2431*	*0.5061*	*0.3197*	*0.3871*
CLU	*100 ± 2.26*	*395.02 ± 42.33*	*164.31 ± 30.71*	*195.75 ± 10.68*	*117.02 ± 16.02*	*176.3 ± 16.98*	*0.1126*	*0.1763*					
AASS	*100 ± 7.72*	*82.97 ± 30.06*	*149.37 ± 3.16*	*75.49 ± 4.79*	*89.89 ± 11.49*	*106.28 ± 10.71*	*0.0089*	*0.0211*	*0.8019*	*0.5254*	*0.9685*	*0.0491*	*0.9960*
PRKCA	*100 ± 16.51*	*95.47 ± 9.29*	*124.36 ± 4.33*	*114.13 ± 8.93*	*125.05 ± 27.8*	*89.56 ± 6.99*	*0.5631*	*0.6231*					
PRKCD	*100 ± 14.51*	*71.96 ± 5.43*	*139.64 ± 4.89*	*135.58 ± 8.19*	*134.46 ± 12.5*	*144.86 ± 8.24*	*0.0018*	*0.0052*					
PRKCE	*100 ± 11.45*	*140.67 ± 7.91*	*121.26 ± 5.06*	*99.58 ± 2.47*	*113.08 ± 10.82*	*120.9 ± 5.49*	*0.0426*	*0.0721*					
PRKCG	*100 ± 7.71*	*94.34 ± 12.69*	*123.81 ± 7.02*	*137.9 ± 6.55*	*105.56 ± 22.63*	*160.53 ± 11.39*	*0.0352*	*0.0648*					
PRKCQ	*100 ± 13.62*	*256.87 ± 28.7*	*83.65 ± 5.42*	*161.16 ± 10.94*	*112.88 ± 25.06*	*130.2 ± 3.47*	*0.0222*	*0.0448*	*0.0162*	*0.5834*	*0.9988*	*0.9964*	*0.9505*
PRKCZ	*100 ± 11.66*	*104.22 ± 10.27*	*119.01 ± 7.41*	*105.07 ± 11.9*	*114.34 ± 5.55*	*119.74 ± 7.13*	*0.6266*	*0.6843*					
ALS2	*100 ± 3.94*	*89 ± 2.58*	*235.42 ± 9.92*	*189.81 ± 8.47*	*144.09 ± 10.06*	*252.76 ± 4.64*	*0.0000*	*0.0000*	*0.9708*	*0.0011*	*0.1385*	*0.0001*	*0.0000*
GNAO1	*100 ± 25.06*	*136.21 ± 3.57*	*77.18 ± 7.81*	*58.92 ± 13.25*	*83.42 ± 22.03*	*87.24 ± 12.09*	*0.0240*	*0.0462*					

GNB1	*100 ± 46.06*	*116 ± 25.57*	*212.07 ± 15.27*	*107 ± 42.55*	*209.8 ± 38.13*	*298.29 ± 4.08*	*0.0392*	*0.0703*					
GNB2	*100 ± 4.42*	*132.14 ± 3.47*	*72.14 ± 4.31*	*79.14 ± 4.84*	*60.6 ± 13.35*	*75.99 ± 4.39*	*0.0000*	*0.0000*	*0.0008*	*0.0284*	*0.0001*	*0.0034*	*0.0108*
EP300	*100 ± 5.39*	*168.04 ± 4.81*	*72.09 ± 5.43*	*76.11 ± 2.91*	*84.24 ± 7.92*	*75.76 ± 5.98*	*0.0000*	*0.0000*	*0.0000*	*0.0256*	*0.1948*	*0.0086*	*0.0232*
HDAC1	*100 ± 14.74*	*116.09 ± 1.79*	*127.66 ± 6.49*	*84.07 ± 12.78*	*112.68 ± 16.11*	*119.04 ± 5.76*	*0.1526*	*0.2302*					
CDKL1	*100 ± 30.06*	*139.3 ± 11.46*	*123.66 ± 24.67*	*99.86 ± 23.64*	*121.66 ± 25.84*	*145.96 ± 24.15*	*0.8041*	*0.8342*					
GAP43	*100 ± 23*	*178.87 ± 7.57*	*89.16 ± 11.31*	*68.57 ± 8.87*	*80.16 ± 17.32*	*89.68 ± 9.5*	*0.0003*	*0.0014*	*0.0029*	*0.3730*	*0.7566*	*0.9696*	*0.9752*
GSK3a	*100 ± 8.41*	*131.06 ± 3.09*	*118.59 ± 7.57*	*99.24 ± 10.15*	*128.19 ± 11.02*	*124.07 ± 6.82*	*0.1023*	*0.1633*					
GSK3b	*100 ± 35.67*	*177.47 ± 14.66*	*146.04 ± 12.41*	*107.17 ± 24.76*	*156.78 ± 20.2*	*214.92 ± 25.99*	*0.2155*	*0.2981*					
APBA1	*100 ± 9.05*	*123.72 ± 13.24*	*100.53 ± 11.8*	*96.21 ± 10.51*	*116.87 ± 17.53*	*95.7 ± 4.12*	*0.5546*	*0.6220*					
APBA3	*100 ± 8.94*	*127.53 ± 6.05*	*84.44 ± 14.18*	*103.32 ± 7.99*	*125.94 ± 11.47*	*104.55 ± 9.56*	*0.0720*	*0.1172*					
APBB1	*100 ± 18.6*	*136.32 ± 4.54*	*139.57 ± 17.3*	*124.1 ± 22.53*	*168.39 ± 26.3*	*171.1 ± 9.41*	*0.3944*	*0.4553*					
APBB2	*100 ± 4.59*	*163.7 ± 6.55*	*89.68 ± 24.61*	*96.23 ± 11.31*	*85.09 ± 4.66*	*88.57 ± 9.41*	*0.0013*	*0.0041*	*0.0050*	*0.9995*	*0.8352*	*0.9546*	*0.9331*
APPBP1	*100 ± 12.44*	*135.96 ± 3.63*	*118.68 ± 14.66*	*102.03 ± 9.13*	*116.2 ± 26.43*	*84.73 ± 3.74*	*0.3263*	*0.4042*					
UBQLN1	*100 ± 12.65*	*138.66 ± 5.91*	*86.23 ± 2.97*	*84.09 ± 3.67*	*99.19 ± 4.13*	*86.92 ± 4.19*	*0.0002*	*0.0008*	*0.0031*	*0.3526*	*1.0000*	*0.4818*	*0.5285*
APH1a	*100 ± 6.61*	*124.11 ± 8.08*	*61.15 ± 3.03*	*54.08 ± 5.42*	*55.78 ± 13.35*	*61.91 ± 7.23*	*0.0000*	*0.0000*	*0.0517*	*0.0003*	*0.0004*	*0.0018*	*0.0018*
APH1b	*100 ± 3.58*	*141.08 ± 8.72*	*61.04 ± 19.27*	*60.08 ± 6.73*	*69.3 ± 4.82*	*62.76 ± 16.81*	*0.0000*	*0.0001*	*0.0135*	*0.0167*	*0.0788*	*0.0197*	*0.0265*
CASP3	*100 ± 13.66*	*172.59 ± 9.92*	*62.93 ± 22.17*	*66.89 ± 6.54*	*89.15 ± 6.86*	*76.32 ± 3.59*	*0.0000*	*0.0001*	*0.0009*	*0.1715*	*0.9321*	*0.1083*	*0.4413*
CTSB	*100 ± 7.97*	*114.94 ± 4.69*	*84.86 ± 10*	*95.01 ± 10.05*	*87.31 ± 18.31*	*64.01 ± 13.49*	*0.0398*	*0.0703*					
CTSL	*100 ± 9.63*	*136.7 ± 7.8*	*64.73 ± 19.38*	*77.65 ± 6.34*	*91.07 ± 11.56*	*61.53 ± 18.35*	*0.0006*	*0.0023*					
PSENEN	*100 ± 7.53*	*143.11 ± 10.9*	*74.08 ± 5.41*	*69.19 ± 5.97*	*100.88 ± 4.38*	*61.31 ± 8.31*	*0.0000*	*0.0001*	*0.0050*	*0.0520*	*1.0000*	*0.1204*	*0.0121*
A2M	*100 ± 13.67*	*69.58 ± 19.04*	*576.6 ± 20.29*	*445.48 ± 13.64*	*169.52 ± 19.46*	*451.01 ± 10.09*	*0.0000*	*0.0001*	*0.9953*	*0.0027*	*0.8703*	*0.0004*	*0.0024*
ECE1	*100 ± 16.03*	*73.71 ± 12.58*	*61.72 ± 22.42*	*112.92 ± 22.37*	*94.85 ± 34.2*	*55.56 ± 35.03*	*0.3459*	*0.4222*					
ECE2	*100 ± 16.17*	*150.04 ± 3.8*	*54.4 ± 13.25*	*60.26 ± 10.37*	*84.74 ± 14.71*	*50.91 ± 6.35*	*0.0000*	*0.0001*	*0.0069*	*0.0353*	*0.6923*	*0.0142*	*0.0082*
LRP1	*100 ± 8.94*	*117.13 ± 12.85*	*50.23 ± 20.72*	*80.94 ± 6.94*	*91.21 ± 9.11*	*72.62 ± 13.45*	*0.0038*	*0.0105*	*0.6415*	*0.5509*	*0.9561*	*0.0110*	*0.2375*
MMP2	*100 ± 15.48*	*132.24 ± 6.29*	*74.3 ± 7.42*	*81.52 ± 12.27*	*63.2 ± 9.62*	*54.8 ± 8.94*	*0.0001*	*0.0007*	*0.0840*	*0.4896*	*0.0420*	*0.2110*	*0.0107*
PKP4	*100 ± 5.41*	*122.49 ± 7.22*	*106.3 ± 4.25*	*105.14 ± 6.88*	*105.1 ± 9.83*	*97.1 ± 2.03*	*0.2094*	*0.2946*					

PRNP	*100 ± 14.59*	*102.2 ± 7.09*	*154.6 ± 9.38*	*159.86 ± 9.45*	*85.13 ± 13.13*	*162.92 ± 9.61*	*0.0009*	*0.0031*	*1.0000*	*0.0219*	*0.8931*	*0.0388*	*0.0155*
SNCA	*100 ± 15.06*	*105.36 ± 4.06*	*101.69 ± 7.8*	*92.43 ± 12.59*	*103.85 ± 19.45*	*63.96 ± 8.07*	*0.1921*	*0.2845*					
SNCB	*100 ± 12.79*	*131.41 ± 20.02*	*58.34 ± 8.74*	*71.52 ± 9.36*	*87.55 ± 20.49*	*51.38 ± 8.15*	*0.0109*	*0.0251*					
CASP4	*100 ± 8.07*	*150.49 ± 6.57*	*80.81 ± 17.27*	*83.26 ± 5.06*	*110.84 ± 5.17*	*86.42 ± 12.92*	*0.0005*	*0.0020*	*0.0056*	*0.6042*	*0.8799*	*0.4840*	*0.7622*
IL1A	*100 ± 5.05*	*119.17 ± 5.31*	*756.46 ± 14.51*	*835.5 ± 15.27*	*102.71 ± 7.19*	*818.49 ± 3.93*	*0.0000*	*0.0000*	*0.9998*	*0.0000*	*1.0000*	*0.0002*	*0.0000*
SH2D3C	*100 ± 18.59*	*121.91 ± 7.12*	*83.8 ± 26.91*	*100.6 ± 16.24*	*119.72 ± 26.2*	*60.19 ± 19.84*	*0.2757*	*0.3691*					
MAP2	*100 ± 8.84*	*97.16 ± 8.22*	*112.66 ± 8.18*	*111.72 ± 12.97*	*116.92 ± 4.44*	*91.82 ± 10.26*	*0.3950*	*0.4553*					
MAPT	*100 ± 4.3*	*119.89 ± 8.16*	*153.39 ± 3*	*141.78 ± 12.13*	*83.47 ± 16.91*	*142.22 ± 6.99*	*0.0017*	*0.0050*	*0.5910*	*0.0589*	*0.7361*	*0.0126*	*0.0555*
PLAU	*100 ± 3.87*	*138.9 ± 6.29*	*94.51 ± 7.63*	*119.94 ± 4.13*	*93.31 ± 4.2*	*78.66 ± 10.44*	*0.0001*	*0.0003*	*0.0020*	*0.1513*	*0.9165*	*0.9604*	*0.1141*
PLAT	*100 ± 14.66*	*135.67 ± 11.1*	*108.59 ± 5.01*	*95.86 ± 5.91*	*99.25 ± 18.03*	*96.1 ± 5.92*	*0.2007*	*0.2871*					
ACHE	*100 ± 14.93*	*125.35 ± 22.22*	*165.64 ± 4.95*	*224.64 ± 8.61*	*130.02 ± 25.38*	*164.85 ± 5.25*	*0.0087*	*0.0211*	*0.8558*	*0.0022*	*0.7624*	*0.1391*	*0.1458*
BDNF	*100 ± 4.13*	*159.65 ± 22.93*	*187.3 ± 19.86*	*183.29 ± 6.71*	*92.58 ± 16.36*	*170.92 ± 7.45*	*0.0298*	*0.0563*					

Supplement S4. Analysis of significant differences between the transcriptional effects of the analyzed methylxanthine variants in SH-SY5Y cells. *P values of ANOVA, adjusted ANOVA and Tukey HSD post-hoc test used for multi comparison analysis are listed.*

gene	p values											
	**ANOVA**	**ANOVA adj.**	**C vs. TP**	**C vs. P**	**C vs. TB**	**C vs. PF**	**TP vs. P**	**TP vs. TB**	**TP vs. PF**	**P vs. TB**	**P vs. PF**	**TB vs. PF**
PARK7	*0.0001*	*0.0002*	*0.0007*	*0.0487*	*0.0067*	*0.0000*	*0.2327*	*0.7715*	*0.5035*	*0.8351*	*0.0122*	*0.0862*
PRDX1	*0.0789*	*0.0834*	*0.6814*	*0.8357*	*0.7077*	*0.6661*	*0.1806*	*0.1200*	*0.1058*	*0.9991*	*0.9976*	*1.0000*
PRDX6	*0.0071*	*0.0093*	*0.9552*	*0.0100*	*0.2059*	*0.0508*	*0.0378*	*0.5281*	*0.1721*	*0.4893*	*0.9106*	*0.9256*
SOD1	*0.0871*	*0.0895*	*0.3401*	*0.6469*	*0.0973*	*0.1098*	*0.9795*	*0.9316*	*0.9499*	*0.6727*	*0.7123*	*1.0000*
TXNIP	*0.0308*	*0.0368*	*0.9235*	*0.1052*	*0.9029*	*1.0000*	*0.0234*	*0.4731*	*0.9385*	*0.4056*	*0.0961*	*0.8838*
TXNRD2	*0.0000*	*0.0001*	*0.0078*	*0.5133*	*0.0004*	*0.0001*	*0.1561*	*0.5728*	*0.1249*	*0.0095*	*0.0012*	*0.8185*
IDH1	*0.0208*	*0.0257*	*0.8051*	*0.7055*	*0.4932*	*0.1454*	*0.1744*	*0.0937*	*0.0194*	*0.9958*	*0.7474*	*0.9125*
ERCC2	*0.0000*	*0.0000*	*0.0000*	*0.0000*	*0.0000*	*0.0000*	*0.3840*	*0.6497*	*0.9832*	*0.9890*	*0.1757*	*0.3562*
ERCC6	*0.0043*	*0.0063*	*0.0209*	*0.0191*	*0.0059*	*0.0089*	*1.0000*	*0.9632*	*0.9914*	*0.9718*	*0.9944*	*0.9995*
ABCA1	*0.0025*	*0.0040*	*0.9995*	*0.0682*	*0.7055*	*0.3836*	*0.0466*	*0.8159*	*0.4947*	*0.0059*	*0.0020*	*0.9779*
APOA1	*0.0028*	*0.0042*	*0.0099*	*0.6224*	*0.0058*	*0.0229*	*0.1409*	*0.9986*	*0.9919*	*0.0877*	*0.2800*	*0.9521*
HADH2	*0.0000*	*0.0000*	*0.0000*	*0.0004*	*0.0000*	*0.0000*	*0.1956*	*0.9409*	*0.9850*	*0.5433*	*0.0818*	*0.7176*
LPL	*0.0454*	*0.0494*	*0.9507*	*0.1884*	*1.0000*	*0.1366*	*0.5072*	*0.9286*	*0.4010*	*0.1647*	*0.9996*	*0.1186*
ERN1	*0.0013*	*0.0024*	*0.0026*	*0.0064*	*0.0035*	*0.0045*	*0.9890*	*0.9998*	*0.9984*	*0.9978*	*0.9997*	*0.9999*
AASS	*0.0104*	*0.0132*	*0.9943*	*0.9957*	*0.0224*	*0.7333*	*0.9377*	*0.0104*	*0.5030*	*0.0449*	*0.9046*	*0.2050*
PRKCQ	*0.0374*	*0.0433*	*0.3759*	*0.0839*	*0.0291*	*0.1508*	*0.8766*	*0.5719*	*0.9724*	*0.9776*	*0.9969*	*0.8899*
ALS2	*0.0000*	*0.0000*	*0.0023*	*0.1287*	*0.0001*	*0.0000*	*0.2597*	*0.2618*	*0.0673*	*0.0054*	*0.0012*	*0.9247*
GNB2	*0.0000*	*0.0000*	*0.0000*	*0.0000*	*0.0000*	*0.0000*	*0.1094*	*0.8507*	*0.9905*	*0.4885*	*0.2306*	*0.9803*
EP300	*0.0000*	*0.0000*	*0.0000*	*0.0000*	*0.0000*	*0.0000*	*0.8293*	*0.9843*	*1.0000*	*0.5397*	*0.8075*	*0.9888*
GAP43	*0.0000*	*0.0001*	*0.0000*	*0.0001*	*0.0003*	*0.0003*	*0.9392*	*0.6702*	*0.6502*	*0.9750*	*0.9694*	*1.0000*
APBB2	*0.0024*	*0.0040*	*0.0139*	*0.0042*	*0.0069*	*0.0061*	*0.9694*	*0.9958*	*0.9923*	*0.9989*	*0.9996*	*1.0000*

UBQLN1	*0.0000*	*0.0000*	*0.0000*	*0.0003*	*0.0000*	*0.0000*	*0.2170*	*0.9975*	*0.9927*	*0.3465*	*0.3977*	*1.0000*
APH1a	*0.0000*	*0.0000*	*0.0000*	*0.0000*	*0.0000*	*0.0000*	*0.9996*	*0.9216*	*0.8915*	*0.9696*	*0.9518*	*1.0000*
APH1b	*0.0001*	*0.0001*	*0.0001*	*0.0005*	*0.0002*	*0.0002*	*0.9524*	*1.0000*	*0.9996*	*0.9677*	*0.9862*	*0.9999*
CASP3	*0.0000*	*0.0000*	*0.0000*	*0.0004*	*0.0000*	*0.0001*	*0.5783*	*0.9988*	*0.9668*	*0.4270*	*0.9057*	*0.8918*
PSENEN	*0.0000*	*0.0001*	*0.0001*	*0.0150*	*0.0002*	*0.0000*	*0.0858*	*0.9920*	*0.9548*	*0.1799*	*0.0235*	*0.7912*
A2M	*0.0002*	*0.0005*	*0.0068*	*0.8049*	*0.0004*	*0.0060*	*0.0555*	*0.6130*	*1.0000*	*0.0035*	*0.0496*	*0.6488*
ECE2	*0.0000*	*0.0000*	*0.0000*	*0.0002*	*0.0000*	*0.0000*	*0.2061*	*0.9809*	*0.9036*	*0.0816*	*0.0449*	*0.9973*
LRP1	*0.0054*	*0.0077*	*0.1466*	*0.4197*	*0.0028*	*0.0531*	*0.9522*	*0.2669*	*0.9774*	*0.0828*	*0.7090*	*0.5562*
MMP2	*0.0000*	*0.0001*	*0.0014*	*0.0001*	*0.0004*	*0.0000*	*0.4120*	*0.9516*	*0.1168*	*0.8105*	*0.9193*	*0.3537*
PRNP	*0.0014*	*0.0024*	*0.0483*	*0.8848*	*0.0812*	*0.0354*	*0.0083*	*0.9984*	*0.9998*	*0.0143*	*0.0060*	*0.9908*
CASP4	*0.0006*	*0.0011*	*0.0015*	*0.0708*	*0.0011*	*0.0023*	*0.3048*	*0.9997*	*0.9993*	*0.2337*	*0.4163*	*0.9934*
IL1A	*0.0000*	*0.0000*	*0.0001*	*0.9999*	*0.0003*	*0.0001*	*0.0001*	*0.9466*	*0.9998*	*0.0002*	*0.0001*	*0.9773*
MAPT	*0.0066*	*0.0091*	*0.6965*	*0.2468*	*0.3182*	*0.6815*	*0.0248*	*0.9559*	*1.0000*	*0.0066*	*0.0236*	*0.9615*
PLAU	*0.0001*	*0.0003*	*0.3340*	*0.0023*	*0.0029*	*0.0001*	*0.0938*	*0.1166*	*0.0053*	*0.9999*	*0.5726*	*0.5005*
ACHE	*0.0392*	*0.0439*	*0.0392*	*0.9999*	*0.6922*	*0.7067*	*0.0521*	*0.3528*	*0.3408*	*0.7761*	*0.7894*	*1.0000*

Supplement S5. Analysis of cell type independent effects. *Detailed overview of pooled transcriptional changes after treatment with caffeine (C), theobromine (TB), theophylline (TP), pentoxifylline (P), and propentofylline (PF) in SH-SY5Y, Calu-3 and HEPG2 cells. Included are the mean expression changes ± standard error of the mean (SEM) after normalization using the five most stable housekeeping genes for each cell line. P values for ANOVA analysis, false discovery rate adjusted p values ("ANOVA adj.") and p values for Dunnett´s Test are shown.*

gene	mean expression changes ± standard error of the mean (SEM) [%]						p value						
	Ctrl	C	TP	P	TB	PF	ANOVA	ANOVA adj.	Crtl vs. C	Ctrl vs. TP	Ctrl vs. P	Ctrl vs. TB	Ctrl vs. PF
PARK7	*100 ± 8.24*	*116.56 ± 12.14*	*99 ± 10.89*	*97.73 ± 16.29*	*101.72 ± 16.71*	*104.61 ± 30.55*	*0.7914*	*0.8186*	*0.6582*	*1.0000*	*0.9999*	*1.0000*	*0.9975*
TXNRD2	*100 ± 5.96*	*116.56 ± 8.58*	*95.73 ± 7.53*	*102.96 ± 15.08*	*77.87 ± 13.85*	*89.87 ± 15*	*0.0047*	*0.0218*	*0.2880*	*0.9891*	*0.9980*	*0.0887*	*0.7285*
ERCC2	*100 ± 6.79*	*128.92 ± 16.78*	*86.36 ± 14.04*	*87.25 ± 14.71*	*87.51 ± 12.75*	*92.87 ± 23.34*	*0.0095*	*0.0259*	*0.0997*	*0.7234*	*0.7706*	*0.7840*	*0.9716*
HADH2	*100 ± 7.92*	*119.48 ± 16.44*	*82.93 ± 6.58*	*101.3 ± 11.6*	*85.37 ± 15.16*	*92.73 ± 15.09*	*0.0104*	*0.0259*	*0.2268*	*0.3408*	*1.0000*	*0.4869*	*0.9315*
ALS2	*100 ± 10.32*	*83.8 ± 10.18*	*115.57 ± 29.88*	*97.89 ± 21.54*	*161.06 ± 40.1*	*151.13 ± 41.45*	*0.0064*	*0.0218*	*0.9339*	*0.9432*	*1.0000*	*0.0449*	*0.1204*
PRDX6	*100 ± 16.31*	*123.1 ± 20.7*	*123.96 ± 20.16*	*113.82 ± 35.93*	*128.07 ± 25.36*	*131.17 ± 31.47*	*0.7120*	*0.7628*	*0.7117*	*0.6828*	*0.9478*	*0.5443*	*0.4448*
GNB2	*100 ± 5.29*	*117.17 ± 7.55*	*95.07 ± 8.48*	*86.41 ± 11.92*	*91.65 ± 8.55*	*97.08 ± 13.77*	*0.0065*	*0.0218*	*0.1255*	*0.9563*	*0.3007*	*0.7355*	*0.9955*
EP300	*100 ± 5.49*	*121.53 ± 19.69*	*82.04 ± 8.25*	*91.15 ± 13.41*	*83.65 ± 10.39*	*74.57 ± 12.9*	*0.0003*	*0.0048*	*0.1446*	*0.2818*	*0.8553*	*0.3666*	*0.0617*
APBB2	*100 ± 5.6*	*118.34 ± 18.96*	*95.65 ± 9.37*	*86.98 ± 9.05*	*87.95 ± 13.6*	*89.55 ± 6.02*	*0.0114*	*0.0263*	*0.1911*	*0.9872*	*0.4963*	*0.5689*	*0.6908*
UBQLN1	*100 ± 13.5*	*114.01 ± 23.29*	*104.85 ± 14.48*	*103.02 ± 32.91*	*95.02 ± 30.61*	*95.28 ± 27.77*	*0.9420*	*0.9420*	*0.9360*	*0.9994*	*0.9999*	*0.9994*	*0.9995*
Casp3	*100 ± 14.65*	*141.37 ± 24.02*	*104.92 ± 18.29*	*97.12 ± 15.65*	*102.29 ± 28.39*	*116.06 ± 27.5*	*0.1510*	*0.2059*	*0.0971*	*0.9989*	*0.9999*	*1.0000*	*0.8421*
PRDX1	*100 ± 10.47*	*131.41 ± 17.42*	*117.96 ± 12.94*	*141.91 ± 16.68*	*136.76 ± 21.53*	*148.56 ± 24.91*	*0.0192*	*0.0411*	*0.1355*	*0.6190*	*0.0249*	*0.0598*	*0.0069*
APH1a	*100 ± 4.46*	*110.16 ± 10.61*	*84.5 ± 12.5*	*97.38 ± 17.12*	*91.53 ± 18.72*	*96.14 ± 17.48*	*0.3844*	*0.4805*	*0.8552*	*0.5483*	*0.9996*	*0.9235*	*0.9974*
A2M	*100 ± 17.02*	*109.19 ± 25.89*	*247.01 ± 82.57*	*152.02 ± 37.69*	*294.07 ±126.18*	*243.94 ± 84.71*	*0.0053*	*0.0218*	*0.9999*	*0.0670*	*0.8541*	*0.0084*	*0.0754*
IL1A	*100 ± 14.21*	*94.12 ± 15.27*	*336.34 ±196.16*	*104.35 ± 17.98*	*359.2 ± 160.33*	*344.56 ±176.88*	*0.0080*	*0.0240*	*1.0000*	*0.0986*	*1.0000*	*0.0591*	*0.0822*
PLAU	*100 ± 10.74*	*169.61 ± 28.83*	*139.77 ± 34.19*	*194.78 ± 60.16*	*143.6 ± 26.4*	*181.85 ± 55.52*	*0.0611*	*0.0965*	*0.1345*	*0.6183*	*0.0211*	*0.5346*	*0.0574*
ACHE	*100 ± 10.01*	*107.03 ± 19.46*	*139.26 ± 35.21*	*133.85 ± 23.36*	*174.95 ± 31.1*	*161.67 ± 22.93*	*0.0025*	*0.0152*	*0.9968*	*0.2089*	*0.3346*	*0.0024*	*0.0156*
AASS	*100 ± 6.59*	*89.54 ± 17.76*	*76.98 ± 12.63*	*92.96 ± 8.17*	*94.07 ± 24.27*	*84.71 ± 10.87*	*0.4871*	*0.5549*	*0.8518*	*0.2090*	*0.9660*	*0.9835*	*0.5803*
ABCA1	*100 ± 9.79*	*99.37 ± 18.59*	*85.36 ± 19.2*	*104.92 ± 8.34*	*77.4 ± 14.87*	*76.7 ± 18.76*	*0.1067*	*0.1600*	*1.0000*	*0.6691*	*0.9945*	*0.2712*	*0.2456*

APH1b	*100 ± 5.72*	*125.04 ± 9.43*	*83.86 ± 11.99*	*89.64 ± 10.36*	*85.08 ± 14.36*	*78.19 ± 13.67*	*0.0000*	*0.0011*	*0.0352*	*0.2860*	*0.6900*	*0.3568*	*0.0823*
APOA1	*100 ± 15.15*	*113.49 ± 34.16*	*173.79 ± 38.88*	*141.78 ± 29.91*	*166.67 ± 38.21*	*157.66 ± 39.43*	*0.0507*	*0.0845*	*0.9841*	*0.0384*	*0.4167*	*0.0721*	*0.1480*
CASP4	*100 ± 7.14*	*108.98 ± 16.81*	*74.87 ± 4.98*	*93.94 ± 8.16*	*83.77 ± 11.26*	*78.12 ± 9.05*	*0.0006*	*0.0058*	*0.7270*	*0.0167*	*0.9236*	*0.2037*	*0.0461*
ERCC6	*100 ± 10.78*	*99.75 ± 16.32*	*66.89 ± 15.84*	*103.51 ± 26.4*	*96.7 ± 28.31*	*108.88 ± 26.05*	*0.2325*	*0.3032*	*1.0000*	*0.2287*	*0.9998*	*0.9998*	*0.9823*
IDH1	*100 ± 11.61*	*104.67 ± 10.12*	*85.05 ± 19.99*	*114.86 ± 21.7*	*133 ± 18.46*	*141.66 ± 22.55*	*0.0025*	*0.0152*	*0.9978*	*0.7650*	*0.7691*	*0.1094*	*0.0263*
LRP1	*100 ± 6.68*	*100.02 ± 12.31*	*87.33 ± 9.17*	*115.96 ± 21.53*	*94.91 ± 26.33*	*103.87 ± 20.64*	*0.4994*	*0.5549*	*1.0000*	*0.8471*	*0.6996*	*0.9963*	*0.9990*
MAPT	*100 ± 11.83*	*97.95 ± 14.92*	*118.84 ± 18.94*	*135.91 ± 41.36*	*153.65 ± 14.46*	*138.59 ± 29.76*	*0.0349*	*0.0649*	*1.0000*	*0.8051*	*0.2550*	*0.0357*	*0.1978*
PRKCQ	*100 ± 10.93*	*166.45 ± 52.62*	*137.86 ± 24.56*	*100.93 ± 29.5*	*101.54 ± 14.95*	*126.8 ± 22.76*	*0.0368*	*0.0649*	*0.0288*	*0.3727*	*1.0000*	*1.0000*	*0.6883*
PRNP	*100 ± 9.72*	*101.97 ± 12.03*	*119.65 ± 22.01*	*96.36 ± 10.86*	*122.5 ± 16.75*	*133.57 ± 17.76*	*0.0231*	*0.0463*	*0.9999*	*0.3936*	*0.9986*	*0.2705*	*0.0408*
PSENEN	*100 ± 6.66*	*113.42 ± 15.6*	*85.31 ± 8.74*	*101.73 ± 11.04*	*92.61 ± 15.87*	*92.91 ± 15.96*	*0.1450*	*0.2059*	*0.5793*	*0.4945*	*0.9999*	*0.9308*	*0.9407*
SOD1	*100 ± 4.15*	*91.52 ± 10.31*	*86.71 ± 9.15*	*84.88 ± 8.72*	*82.55 ± 14.11*	*90.15 ± 15.12*	*0.4415*	*0.5298*	*0.8032*	*0.4308*	*0.3106*	*0.1926*	*0.7004*

Supplement S6. Loading plots corresponding to the principal component analysis.

Loading plot: oxidative stress related genes PC1

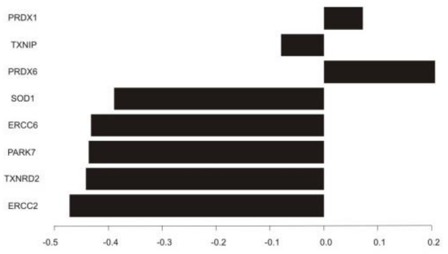

Loading plot: oxidative stress related genes PC2

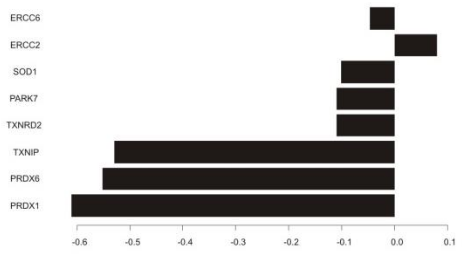

Loading plot: Lipid- & energy-metabolism related genes PC1

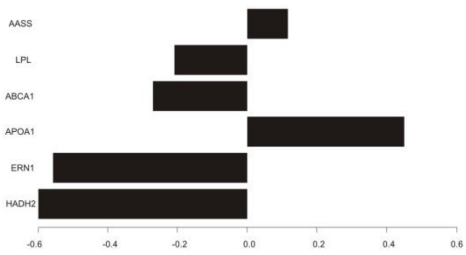

Loading plot: Lipid- & energy-metabolism related genes PC2

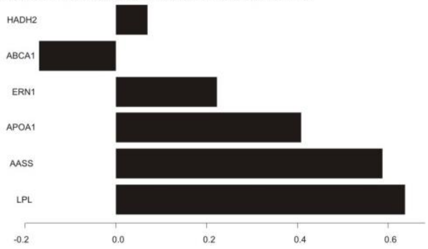

Loading plot: signal transduction and gene expression related genes PC1

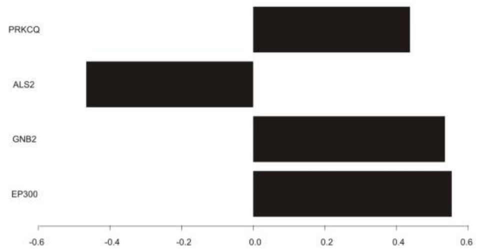

Loading plot: signal transduction and gene expression related genes PC2

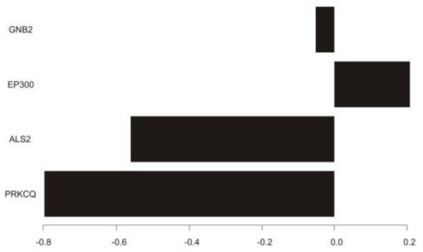

Loading plot: Abeta & tau- pathology & inflammation related genes PC1

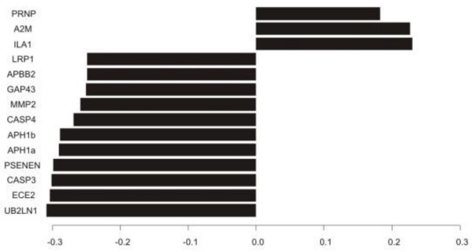

Loading plot: Abeta & tau- pathology & inflammation related genes PC2

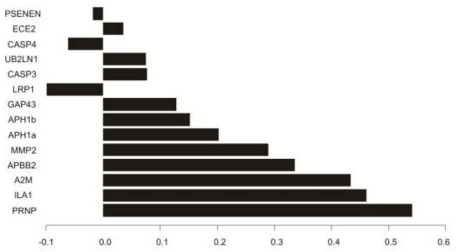

Loading plot: neuronal genes related genes PC1

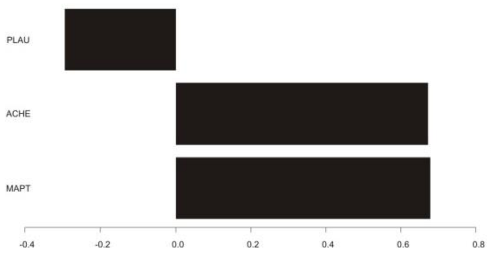

Loading plot: neuronal genes related genes PC2

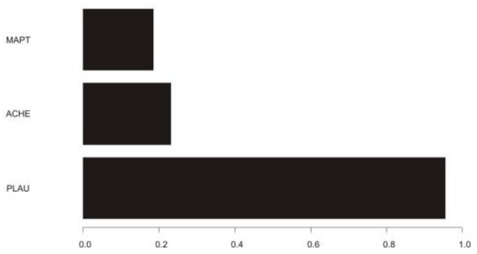

References

1. Nandi, A.; Yan, L.J.; Jana, C.K.; Das, N. Role of catalase in oxidative stress- and age-associated degenerative diseases. *Oxidative medicine and cellular longevity* **2019**, *2019*, 9613090.
2. Maksimenko, A.V.; Vavaev, A.V. Antioxidant enzymes as potential targets in cardioprotection and treatment of cardiovascular diseases. Enzyme antioxidants: The next stage of pharmacological counterwork to the oxidative stress. *Heart international* **2012**, *7*, e3.
3. Bajpai, J.; Prakash, V.; Kant, S.; Verma, A.K.; Srivastava, A.; Bajaj, D.K.; Ahmad, M.K.; Agarwal, A. Study of oxidative stress biomarkers in chronic obstructive pulmonary disease and their correlation with disease severity in north indian population cohort. *Lung India : official organ of Indian Chest Society* **2017**, *34*, 324-329.
4. Kasapoglu, M.; Ozben, T. Alterations of antioxidant enzymes and oxidative stress markers in aging. *Experimental gerontology* **2001**, *36*, 209-220.
5. Rinaldi, P.; Polidori, M.C.; Metastasio, A.; Mariani, E.; Mattioli, P.; Cherubini, A.; Catani, M.; R.; Senin, U.; Mecocci, P. Plasma antioxidants are similarly depleted in mild cognitive impairment in alzheimer's disease. *Neurobiology of aging* **2003**, *24*, 915-919.
6. Steyn, M.; Zitouni, K.; Kelly, F.J.; Cook, P.; Earle, K.A. Sex differences in glutathione peroxidase activity and central obesity in patients with type 2 diabetes at high risk of cardio-renal disease. *Antioxidants* **2019**, *8*.
7. Joppa, P.; Petrasova, D.; Stancak, B.; Dorkova, Z.; Tkacova, R. Oxidative stress in patients with copd and pulmonary hypertension. *Wiener klinische Wochenschrift* **2007**, *119*, 428-434.
8. Ma, Q.L.; Yang, J.F.; Shao, M.; Dong, X.M.; Chen, B. [association between nad(p)h: Quinone oxidoreductase and apolipoprotein e gene polymorphisms in alzheimer's disease]. *Zhonghua yi xue za zhi* **2003**, *83*, 2124-2127.
9. Chhetri, J.; King, A.E.; Gueven, N. Alzheimer's disease and nqo1: Is there a link? *Current Alzheimer research* **2018**, *15*, 56-66.
10. Boroumand, M.; Pourgholi, L.; Goodarzynejad, H.; Ziaee, S.; Hajhosseini-Talasaz, A.; Sotoudeh-Anvari, M.; Mandegary, A. Nqo1 c609t polymorphism is associated with coronary artery disease in a gender-dependent manner. *Cardiovascular toxicology* **2017**, *17*, 35-41.
11. An, Y.; Feng, L.; Zhang, X.; Wang, Y.; Wang, Y.; Tao, L.; Qin, Z.; Xiao, R. Dietary intakes and patterns of folate, vitamin b6, and vitamin b12 can be associated with cognitive impairment by hypermethylation of redox-related genes nudt15 and txnrd1. *Clinical epigenetics* **2019**, *11*, 139.
12. Baulac, S.; Lu, H.; Strahle, J.; Yang, T.; Goldberg, M.S.; Shen, J.; Schlossmacher, M.G.; Lemere, C.A.; Q.; Xia, W. Increased dj-1 expression under oxidative stress and in alzheimer's disease brains. *Molecular neurodegeneration* **2009**, *4*, 12.
13. Won, K.J.; Jung, S.H.; Jung, S.H.; Lee, K.P.; Lee, H.M.; Lee, D.Y.; Park, E.S.; Kim, J.; Kim, B. Dj-1/park7 modulates vasorelaxation and blood pressure via epigenetic modification of endothelial nitric oxide synthase. *Cardiovascular research* **2014**, *101*, 473-481.
14. Sandford, A.J.; Malhotra, D.; Boezen, H.M.; Siedlinski, M.; Postma, D.S.; Wong, V.; Akhabir, L.; He, J.Q.; Connett, J.E.; Anthonisen, N.R., *et al.* Nfe2l2 pathway polymorphisms and lung function decline in chronic obstructive pulmonary disease. *Physiological genomics* **2012**, *44*, 754-763.

15. Somaio Neto, F.; Ikejiri, A.T.; Bertoletto, P.R.; Chaves, J.C.; Teruya, R.; Fagundes, D.J.; Taha, M.O. expression related to oxidative stress in the heart of mice after intestinal ischemia. *Arquivos brasileiros cardiologia* **2014**, *102*, 165-173.
16. Krzyzosiak, A.; Sigurdardottir, A.; Luh, L.; Carrara, M.; Das, I.; Schneider, K.; Bertolotti, A. Target-based discovery of an inhibitor of the regulatory phosphatase ppp1r15b. *Cell* **2018**, *174*, 1216-1228 e1219.
17. El Eter, E.; Al-Masri, A.A. Peroxiredoxin isoforms are associated with cardiovascular risk factors in type 2 diabetes mellitus. *Brazilian journal of medical and biological research = Revista brasileira de pesquisas medicas e biologicas* **2015**, *48*, 465-469.
18. Pierrou, S.; Broberg, P.; O'Donnell, R.A.; Pawlowski, K.; Virtala, R.; Lindqvist, E.; Richter, A.; Wilson, S.J.; Angco, G.; Moller, S., *et al.* Expression of genes involved in oxidative stress responses in airway epithelial cells of smokers with chronic obstructive pulmonary disease. *American journal of respiratory and critical care medicine* **2007**, *175*, 577-586.
19. Murakami, K.; Murata, N.; Noda, Y.; Tahara, S.; Kaneko, T.; Kinoshita, N.; Hatsuta, H.; Murayama, S.; Barnham, K.J.; Irie, K., *et al.* Sod1 (copper/zinc superoxide dismutase) deficiency drives amyloid beta protein oligomerization and memory loss in mouse model of alzheimer disease. *The Journal of biological chemistry* **2011**, *286*, 44557-44568.
20. Otaki, Y.; Watanabe, T.; Nishiyama, S.; Takahashi, H.; Arimoto, T.; Shishido, T.; Miyamoto, T.; Konta, T.; Shibata, Y.; Sato, H., *et al.* The impact of superoxide dismutase-1 genetic variation on and all-cause mortality in a prospective cohort study: The yamagata (takahata) study. *PLoS One* **2016**, *11*, e0164732.
21. Lewandowski, L.; Kepinska, M.; Milnerowicz, H. The copper-zinc superoxide dismutase activity in selected diseases. *European journal of clinical investigation* **2019**, *49*, e13036.
22. Perrone, L.; Sbai, O.; Nawroth, P.P.; Bierhaus, A. The complexity of sporadic alzheimer's disease pathogenesis: The role of rage as therapeutic target to promote neuroprotection by inhibiting neurovascular dysfunction. *International journal of Alzheimer's disease* **2012**, *2012*, 734956.
23. Chong, C.R.; Chan, W.P.; Nguyen, T.H.; Liu, S.; Procter, N.E.; Ngo, D.T.; Sverdlov, A.L.; Chirkov, Horowitz, J.D. Thioredoxin-interacting protein: Pathophysiology and emerging in cardiovascular disease and diabetes. *Cardiovascular drugs and therapy* **2014**, *28*, 347-360.
24. Matsumura, K.; Ito, S. Novel biomarker genes which distinguish between smokers and chronic obstructive pulmonary disease patients with machine learning approach. *BMC pulmonary medicine* **2020**, *20*, 29.
25. Kiermayer, C.; Northrup, E.; Schrewe, A.; Walch, A.; de Angelis, M.H.; Schoensiegel, F.; Zischka, H.; Prehn, C.; Adamski, J.; Bekeredjian, R., *et al.* Heart-specific knockout of the mitochondrial thioredoxin reductase (txnrd2) induces metabolic and contractile dysfunction in the aging myocardium. *Journal of the American Heart Association* **2015**, *4*.
26. Elko, E.A.; Mahoney, J.M.; Vacek, P.; van der Vliet, A.; Anathy, V.; van der Velden, J.; Janssen-Heininger, Y.M.W.; Seward, D.J. Age-dependent dysregulation of redox genes may to fibrotic pulmonary disease susceptibility. *Free radical biology & medicine* **2019**, *141*, 438-446.
27. Pascual-Gamarra, J.M.; Salazar-Tortosa, D.F.; Labayen, I.; Ruperez, A.I.; Leclercq, C.; Marcos, A.; Gomez, S.; Moreno, L.A.; Meirhaeghe, A.; Castillo, M.J., *et al.* Association of ucp1, ucp2 and ucp3 gene polymorphisms with cardiovascular disease risk factors in european adolescents: The helena study. *Pediatric research* **2020**, *88*, 265-270.

28. Edwards, K.S.; Ashraf, S.; Lomax, T.M.; Wiseman, J.M.; Hall, M.E.; Gava, F.N.; Hall, J.E.; Hosler, J.P.; Harmancey, R. Uncoupling protein 3 deficiency impairs myocardial fatty acid oxidation and contractile recovery following ischemia/reperfusion. *Basic research in cardiology* **2018**, *113*, 47.
29. Gosker, H.R.; Schrauwen, P.; Hesselink, M.K.; Schaart, G.; van der Vusse, G.J.; Wouters, E.F.; Schols, A.M. Uncoupling protein-3 content is decreased in peripheral skeletal muscle of patients with copd. *The European respiratory journal* **2003**, *22*, 88-93.
30. Yi, T.; Wu, X.; Long, Z.; Duan, G.; Wu, Z.; Li, H.; Chen, H.; Zhou, X. Overexpression of ubiquinol-cytochrome c reductase core protein 1 may protect h9c2 cardiac cells by binding with zinc. *BioMed research international* **2017**, *2017*, 1314297.
31. Duan, Y.; Zhou, M.; Xiao, J.; Wu, C.; Zhou, L.; Zhou, F.; Du, C.; Song, Y. Prediction of key genes and mirnas responsible for loss of muscle force in patients during an acute exacerbation of chronic obstructive pulmonary disease. *International journal of molecular medicine* **2016**, *38*, 1450-1462.
32. Ait-Aissa, K.; Blaszak, S.C.; Beutner, G.; Tsaih, S.W.; Morgan, G.; Santos, J.H.; Flister, M.J.; Joyce, D.L.; Camara, A.K.S.; Gutterman, D.D., *et al.* Mitochondrial oxidative phosphorylation defect in the heart of subjects with coronary artery disease. *Scientific reports* **2019**, *9*, 7623.
33. Kang, M.J.; Yoon, C.M.; Kim, B.H.; Lee, C.M.; Zhou, Y.; Sauler, M.; Homer, R.; Dhamija, A.; Boffa, D.; West, A.P., *et al.* Suppression of nlrx1 in chronic obstructive pulmonary disease. *The Journal of clinical investigation* **2015**, *125*, 2458-2462.
34. Liu, S.L.; Wang, C.; Jiang, T.; Tan, L.; Xing, A.; Yu, J.T. The role of cdk5 in alzheimer's disease. *neurobiology* **2016**, *53*, 4328-4342.
35. Strocchi, P.; Pession, A.; Dozza, B. Up-regulation of cdk5/p35 by oxidative stress in human neuroblastoma imr-32 cells. *Journal of cellular biochemistry* **2003**, *88*, 758-765.
36. Arif, A. Extraneuronal activities and regulatory mechanisms of the atypical cyclin-dependent kinase cdk5. *Biochemical pharmacology* **2012**, *84*, 985-993.
37. Bedard, K.; Krause, K.H. The nox family of ros-generating nadph oxidases: Physiology and pathophysiology. *Physiological reviews* **2007**, *87*, 245-313.
38. Barua, S.; Kim, J.Y.; Yenari, M.A.; Lee, J.E. The role of nox inhibitors in neurodegenerative diseases. *IBRO reports* **2019**, *7*, 59-69.
39. Chang, S.; Linderholm, A.; Franzi, L.; Kenyon, N.; Grasberger, H.; Harper, R. Dual oxidase regulates neutrophil recruitment in allergic airways. *Free radical biology & medicine* **2013**, *65*, 38-46.
40. Brown, S.M.; Upadhya, R.; Shoemaker, J.D.; Lodge, J.K. Isocitrate dehydrogenase is important for nitrosative stress resistance in cryptococcus neoformans, but oxidative stress resistance is not dependent on glucose-6-phosphate dehydrogenase. *Eukaryotic cell* **2010**, *9*, 971-980.
41. Szutowicz, A.; Bielarczyk, H.; Zysk, M.; Dys, A.; Ronowska, A.; Gul-Hinc, S.; Klimaszewska-Lata, J. Early and late pathomechanisms in alzheimer's disease: From zinc to amyloid-beta neurotoxicity. *Neurochemical research* **2017**, *42*, 891-904.
42. Hermon, M.; Cairns, N.; Egly, J.M.; Fery, A.; Labudova, O.; Lubec, G. Expression of DNA excision-repair-cross-complementing proteins p80 and p89 in brain of patients with down syndrome and alzheimer's disease. *Neuroscience letters* **1998**, *251*, 45-48.
43. Durik, M.; Kavousi, M.; van der Pluijm, I.; Isaacs, A.; Cheng, C.; Verdonk, K.; Loot, A.E.; Oeseburg, Bhaggoe, U.M.; Leijten, F., *et al.* Nucleotide excision DNA repair is associated with age-related dysfunction. *Circulation* **2012**, *126*, 468-478.

44. Lerner, L.K.; Moreno, N.C.; Rocha, C.R.R.; Munford, V.; Santos, V.; Soltys, D.T.; Garcia, C.C.M.; Sarasin, A.; Menck, C.F.M. Xpd/ercc2 mutations interfere in cellular responses to oxidative stress. *Mutagenesis* **2019**, *34*, 341-354.
45. Gerez, L.; de Haan, A.; Hol, E.M.; Fischer, D.F.; van Leeuwen, F.W.; van Steeg, H.; Benne, R. misreading: The frequency of dinucleotide deletions in neuronal mrnas for beta-amyloid precursor protein and ubiquitin b. *Neurobiology of aging* **2005**, *26*, 145-155.
46. Shah, N.; Meira, L.B.; Elliott, R.M.; Hoole, S.P.; West, N.E.; Brown, A.J.; Bennett, M.R.; Garcia-Garcia, H.M.; Kuku, K.O.; Dan, K., *et al.* DNA damage and repair in patients with coronary artery disease: Correlation with plaque morphology using optical coherence tomography (decode study). *Cardiovascular revascularization medicine : including molecular interventions* **2019**, *20*, 812-818.
47. Adcock, I.M.; Mumby, S.; Caramori, G. Breaking news: DNA damage and repair pathways in copd and implications for pathogenesis and treatment. *The European respiratory journal* **2018**, *52*.
48. Koldamova, R.; Lefterov, I. Role of lxr and abca1 in the pathogenesis of alzheimer's disease - implications for a new therapeutic approach. *Current Alzheimer research* **2007**, *4*, 171-178.
49. Akao, H.; Polisecki, E.; Schaefer, E.J.; Trompet, S.; Robertson, M.; Ford, I.; Jukema, J.W.; de Craen, A.J.; Packard, C.; Buckley, B.M., *et al.* Abca1 gene variation and heart disease risk reduction in the elderly during pravastatin treatment. *Atherosclerosis* **2014**, *235*, 176-181.
50. Oram, J.F. Abca1 as a new therapeutic target for treating cardiovascular disease. *Drug news & perspectives* **2002**, *15*, 24-28.
51. Chai, A.B.; Ammit, A.J.; Gelissen, I.C. Examining the role of abc lipid transporters in pulmonary lipid homeostasis and inflammation. *Respiratory research* **2017**, *18*, 41.
52. Bryleva, E.Y.; Rogers, M.A.; Chang, C.C.; Buen, F.; Harris, B.T.; Rousselet, E.; Seidah, N.G.; Oddo, S.; LaFerla, F.M.; Spencer, T.A., *et al.* Acat1 gene ablation increases 24(s)-hydroxycholesterol content in brain and ameliorates amyloid pathology in mice with ad. *Proc Natl Acad Sci U S A* **2010**, *107*, 3081-3086.
53. Shibuya, Y.; Chang, C.C.; Chang, T.Y. Acat1/soat1 as a therapeutic target for alzheimer's disease. *Future medicinal chemistry* **2015**, *7*, 2451-2467.
54. Wang, Y.T.; Wang, Y.H.; Ma, Y.T.; Fu, Z.Y.; Yang, Y.N.; Ma, X.; Li, X.M.; Adi, D.; Liu, F.; Chen, B.D. Acat-1 gene polymorphism is associated with increased susceptibility to coronary artery disease in chinese han population: A case-control study. *Oncotarget* **2017**, *8*, 89055-89063.
55. Kim, J.H.; Ee, S.M.; Jittiwat, J.; Ong, E.S.; Farooqui, A.A.; Jenner, A.M.; Ong, W.Y. Increased of acyl-coenzyme a: Cholesterol acyltransferase-1 and elevated cholesteryl esters in the hippocampus after excitotoxic injury. *Neuroscience* **2011**, *185*, 125-134.
56. Koldamova, R.P.; Lefterov, I.M.; Lefterova, M.I.; Lazo, J.S. Apolipoprotein a-i directly interacts with amyloid precursor protein and inhibits a beta aggregation and toxicity. *Biochemistry* **2001**, *40*, 3553-3560.
57. Casserly, I.; Topol, E. Convergence of atherosclerosis and alzheimer's disease: Inflammation, cholesterol, and misfolded proteins. *Lancet* **2004**, *363*, 1139-1146.
58. Emoto, T.; Sawada, T.; Morimoto, N.; Tenjin, T.; Wakimoto, T.; Ikeda, F.; Sato, C.; Terashita, D.; Mizoguchi, T.; Mizuguchi, T., *et al.* The apolipoprotein b/a1 ratio is associated with reactive oxygen metabolites and endothelial dysfunction in statin-treated patients with coronary artery disease. *of atherosclerosis and thrombosis* **2013**, *20*, 623-629.

59. Gordon, E.M.; Figueroa, D.M.; Barochia, A.V.; Yao, X.; Levine, S.J. High-density lipoproteins and apolipoprotein a-i: Potential new players in the prevention and treatment of lung disease. *Frontiers in pharmacology* **2016**, *7*, 323.
60. Kim, C.; Lee, J.M.; Park, S.W.; Kim, K.S.; Lee, M.W.; Paik, S.; Jang, A.S.; Kim, D.J.; Uh, S.; Kim, Y., *et al.* Attenuation of cigarette smoke-induced emphysema in mice by apolipoprotein a-1 overexpression. *American journal of respiratory cell and molecular biology* **2016**, *54*, 91-102.
61. Recuero, M.; Vicente, M.C.; Martinez-Garcia, A.; Ramos, M.C.; Carmona-Saez, P.; Sastre, I.; Aldudo, J.; Vilella, E.; Frank, A.; Bullido, M.J., *et al.* A free radical-generating system induces the cholesterol biosynthesis pathway: A role in alzheimer's disease. *Aging cell* **2009**, *8*, 128-139.
62. Ference, B.A.; Robinson, J.G.; Brook, R.D.; Catapano, A.L.; Chapman, M.J.; Neff, D.R.; Voros, S.; Giugliano, R.P.; Davey Smith, G.; Fazio, S., *et al.* Variation in pcsk9 and hmgcr and risk of cardiovascular disease and diabetes. *The New England journal of medicine* **2016**, *375*, 2144-2153.
63. Namba, Y.; Tomonaga, M.; Kawasaki, H.; Otomo, E.; Ikeda, K. Apolipoprotein e immunoreactivity in cerebral amyloid deposits and neurofibrillary tangles in alzheimer's disease and kuru plaque amyloid in creutzfeldt-jakob disease. *Brain research* **1991**, *541*, 163-166.
64. Yao, X.; Gordon, E.M.; Figueroa, D.M.; Barochia, A.V.; Levine, S.J. Emerging roles of apolipoprotein e and apolipoprotein a-i in the pathogenesis and treatment of lung disease. *American journal of cell and molecular biology* **2016**, *55*, 159-169.
65. Mahley, R.W. Apolipoprotein e: From cardiovascular disease to neurodegenerative disorders. *Journal of molecular medicine* **2016**, *94*, 739-746.
66. Shea, T.B.; Rogers, E.; Ashline, D.; Ortiz, D.; Sheu, M.S. Apolipoprotein e deficiency promotes increased oxidative stress and compensatory increases in antioxidants in brain tissue. *Free radical biology & medicine* **2002**, *33*, 1115-1120.
67. Schmidt, M.; Benek, O.; Vinklarova, L.; Hrabinova, M.; Zemanova, L.; Chribek, M.; Kralova, V.; L.; Dolezal, R.; Lycka, A., *et al.* Benzothiazolyl ureas are low micromolar and uncompetitive inhibitors of 17beta-hsd10 with implications to alzheimer's disease treatment. *International journal of molecular sciences* **2020**, *21*.
68. Joiner, D.M.; Ke, J.; Zhong, Z.; Xu, H.E.; Williams, B.O. Lrp5 and lrp6 in development and disease. *Trends in endocrinology and metabolism: TEM* **2013**, *24*, 31-39.
69. Ren, L.; Ren, X. Meta-analyses of four polymorphisms of lipoprotein lipase associated with the risk of alzheimer's disease. *Neuroscience letters* **2016**, *619*, 73-78.
70. Rebeck, G.W.; Harr, S.D.; Strickland, D.K.; Hyman, B.T. Multiple, diverse senile plaque-associated proteins are ligands of an apolipoprotein e receptor, the alpha 2-macroglobulin receptor/low-density-lipoprotein receptor-related protein. *Annals of neurology* **1995**, *37*, 211-217.
71. Gong, H.; Dong, W.; Rostad, S.W.; Marcovina, S.M.; Albers, J.J.; Brunzell, J.D.; Vuletic, S. Lipoprotein lipase (lpl) is associated with neurite pathology and its levels are markedly reduced in the dentate gyrus of alzheimer's disease brains. *The journal of histochemistry and cytochemistry : official journal of the Histochemistry Society* **2013**, *61*, 857-868.
72. Geldenhuys, W.J.; Lin, L.; Darvesh, A.S.; Sadana, P. Emerging strategies of targeting lipoprotein lipase for metabolic and cardiovascular diseases. *Drug discovery today* **2017**, *22*, 352-365.
73. da Costa, I.B.; de Labio, R.W.; Rasmussen, L.T.; Viani, G.A.; Chen, E.; Villares, J.; Turecki, G.; Smith, M.A.C.; Payao, S.L.M. Change in insr, apba2 and ide gene expressions in brains of alzheimer's disease patients. *Current Alzheimer research* **2017**, *14*, 760-765.

74. Galkina, E.V.; Butcher, M.; Keller, S.R.; Goff, M.; Bruce, A.; Pei, H.; Sarembock, I.J.; Sanders, J.M.; Nagelin, M.H.; Srinivasan, S., *et al.* Accelerated atherosclerosis in apoe-/- mice heterozygous for the insulin receptor and the insulin receptor substrate-1. *Arteriosclerosis, thrombosis, and vascular biology* **2012**, *32*, 247-256.
75. Tsai, M.J.; Tsai, Y.C.; Chang, W.A.; Lin, Y.S.; Tsai, P.H.; Sheu, C.C.; Kuo, P.L.; Hsu, Y.L. Deducting microrna-mediated changes common in bronchial epithelial cells of asthma and chronic obstructive pulmonary disease-a next-generation sequencing-guided bioinformatic approach. *International journal of molecular sciences* **2019**, *20*.
76. Carling, D. The amp-activated protein kinase cascade--a unifying system for energy control. *Trends in biochemical sciences* **2004**, *29*, 18-24.
77. Yang, Q.; Xu, J.; Ma, Q.; Liu, Z.; Sudhahar, V.; Cao, Y.; Wang, L.; Zeng, X.; Zhou, Y.; Zhang, M., *et al.* Prkaa1/ampkalpha1-driven glycolysis in endothelial cells exposed to disturbed flow protects against atherosclerosis. *Nature communications* **2018**, *9*, 4667.
78. Wu, M.; Wu, Y.; Huang, J.; Wu, Y.; Wu, H.; Jiang, B.; Zhuang, J. Protein expression profile changes of lung tissue in patients with pulmonary hypertension. *PeerJ* **2020**, *8*, e8153.
79. Pedros, I.; Petrov, D.; Allgaier, M.; Sureda, F.; Barroso, E.; Beas-Zarate, C.; Auladell, C.; Pallas, M.; Vazquez-Carrera, M.; Casadesus, G., *et al.* Early alterations in energy metabolism in the hippocampus of appswe/ps1de9 mouse model of alzheimer's disease. *Biochimica et biophysica acta* **2014**, *1842*, 1556-1566.
80. Duran-Aniotz, C.; Cornejo, V.H.; Espinoza, S.; Ardiles, A.O.; Medinas, D.B.; Salazar, C.; Foley, A.; Gajardo, I.; Thielen, P.; Iwawaki, T., *et al.* Ire1 signaling exacerbates alzheimer's disease pathogenesis. *Acta neuropathologica* **2017**, *134*, 489-506.
81. Wang, Z.V.; Hill, J.A. Protein quality control and metabolism: Bidirectional control in the heart. *Cell metabolism* **2015**, *21*, 215-226.
82. Trougakos, I.P.; Gonos, E.S. Chapter 9: Oxidative stress in malignant progression: The role of a sensitive cellular biosensor of free radicals. *Advances in cancer research* **2009**, *104*, 171-210.
83. Foster, E.M.; Dangla-Valls, A.; Lovestone, S.; Ribe, E.M.; Buckley, N.J. Clusterin in alzheimer's disease: Mechanisms, genetics, and lessons from other pathologies. *Frontiers in neuroscience* **2019**, *13*, 164.
84. Dalle-Donne, I.; Giustarini, D.; Colombo, R.; Rossi, R.; Milzani, A. Protein carbonylation in human diseases. *Trends in molecular medicine* **2003**, *9*, 169-176.
85. Lammich, S.; Kojro, E.; Postina, R.; Gilbert, S.; Pfeiffer, R.; Jasionowski, M.; Haass, C.; Fahrenholz, F. Constitutive and regulated alpha-secretase cleavage of alzheimer's amyloid precursor protein by a disintegrin metalloprotease. *Proc Natl Acad Sci U S A* **1999**, *96*, 3922-3927.
86. Alfonso, S.I.; Callender, J.A.; Hooli, B.; Antal, C.E.; Mullin, K.; Sherman, M.A.; Lesne, S.E.; Leitges, M.; Newton, A.C.; Tanzi, R.E., *et al.* Gain-of-function mutations in protein kinase calpha (pkcalpha) may promote synaptic defects in alzheimer's disease. *Science signaling* **2016**, *9*, ra47.
87. Singh, R.M.; Cummings, E.; Pantos, C.; Singh, J. Protein kinase c and cardiac dysfunction: A review. *Heart failure reviews* **2017**, *22*, 843-859.
88. Wang, J.; Sun, L.; Nie, Y.; Duan, S.; Zhang, T.; Wang, W.; Ye, R.D.; Hou, S.; Qian, F. Protein kinase c delta (pkcdelta) attenuates bleomycin induced pulmonary fibrosis via inhibiting nf-kappab signaling pathway. *Frontiers in physiology* **2020**, *11*, 367.
89. Obeidat, M.; Nie, Y.; Fishbane, N.; Li, X.; Bosse, Y.; Joubert, P.; Nickle, D.C.; Hao, K.; Postma, D.S.; Timens, W., *et al.* Integrative genomics of emphysema-associated genes reveals potential disease biomarkers. *American journal of respiratory cell and molecular biology* **2017**, *57*, 411-418.

90. Favit, A.; Grimaldi, M.; Nelson, T.J.; Alkon, D.L. Alzheimer's-specific effects of soluble beta-amyloid on protein kinase c-alpha and -gamma degradation in human fibroblasts. *Proc Natl Acad Sci U S A* *95*, 5562-5567.

91. Willis, C.L.; Meske, D.S.; Davis, T.P. Protein kinase c activation modulates reversible increase in cortical blood-brain barrier permeability and tight junction protein expression during hypoxia and posthypoxic reoxygenation. *Journal of cerebral blood flow and metabolism : official journal of the International Society of Cerebral Blood Flow and Metabolism* **2010**, *30*, 1847-1859.

92. Salek-Ardakani, S.; So, T.; Halteman, B.S.; Altman, A.; Croft, M. Differential regulation of th2 and th1 lung inflammatory responses by protein kinase c theta. *Journal of immunology* **2004**, *173*, 6440-6447.

93. Zaveri, H.P.; Beck, T.F.; Hernandez-Garcia, A.; Shelly, K.E.; Montgomery, T.; van Haeringen, A.; Anderlid, B.M.; Patel, C.; Goel, H.; Houge, G., *et al.* Identification of critical regions and candidate genes for cardiovascular malformations and cardiomyopathy associated with deletions of chromosome 1p36. *PLoS One* **2014**, *9*, e85600.

94. Wu, X.; Sun, X.; Chen, C.; Bai, C.; Wang, X. Dynamic gene expressions of peripheral blood mononuclear cells in patients with acute exacerbation of chronic obstructive pulmonary disease: A preliminary study. *Critical care* **2014**, *18*, 508.

95. Kanekura, K.; Hashimoto, Y.; Niikura, T.; Aiso, S.; Matsuoka, M.; Nishimoto, I. Alsin, the product of als2 gene, suppresses sod1 mutant neurotoxicity through rhogef domain by interacting with sod1 mutants. *The Journal of biological chemistry* **2004**, *279*, 19247-19256.

96. Cai, H.; Lin, X.; Xie, C.; Laird, F.M.; Lai, C.; Wen, H.; Chiang, H.C.; Shim, H.; Farah, M.H.; Hoke, A., *et al.* Loss of als2 function is insufficient to trigger motor neuron degeneration in knock-out mice but predisposes neurons to oxidative stress. *The Journal of neuroscience : the official journal of the Society for Neuroscience* **2005**, *25*, 7567-7574.

97. Smine, A.; Xu, X.; Nishiyama, K.; Katada, T.; Gambetti, P.; Yadav, S.P.; Wu, X.; Shi, Y.C.; Yasuhara, S.; Homburger, V., *et al.* Regulation of brain g-protein go by alzheimer's disease gene presenilin-1. *The Journal of biological chemistry* **1998**, *273*, 16281-16288.

98. Thathiah, A.; De Strooper, B. The role of g protein-coupled receptors in the pathology of alzheimer's disease. *Nature reviews. Neuroscience* **2011**, *12*, 73-87.

99. Evora, P.R.; Nobre, F. The role of g-proteins in the pathophysiology of the cardiovascular diseases. *Arquivos brasileiros de cardiologia* **1999**, *72*, 209-229.

100. Aubry, S.; Shin, W.; Crary, J.F.; Lefort, R.; Qureshi, Y.H.; Lefebvre, C.; Califano, A.; Shelanski, M.L. Assembly and interrogation of alzheimer's disease genetic networks reveal novel regulators of progression. *PLoS One* **2015**, *10*, e0120352.

101. Lu, X.; Deng, Y.; Yu, D.; Cao, H.; Wang, L.; Liu, L.; Yu, C.; Zhang, Y.; Guo, X.; Yu, G. Histone acetyltransferase p300 mediates histone acetylation of ps1 and bace1 in a cellular model of alzheimer's disease. *PLoS One* **2014**, *9*, e103067.

102. Liao, J.; Chen, Z.; He, Q.; Liu, Y.; Wang, J. Differential gene expression analysis and network construction of recurrent cardiovascular events. *Molecular medicine reports* **2016**, *13*, 1746-1764.

103. Ali Bayram, F.A., Ahmet Ozer & Remzi Yigiter. Decreased hdac1 gene expression in patients with alzheimer's disease. *International Journal of Human Genetics* **2014**, *14*, 177-182.

104. Ito, K.; Ito, M.; Elliott, W.M.; Cosio, B.; Caramori, G.; Kon, O.M.; Barczyk, A.; Hayashi, S.; Adcock, Hogg, J.C., *et al.* Decreased histone deacetylase activity in chronic obstructive pulmonary disease. *The New England journal of medicine* **2005**, *352*, 1967-1976.

105. Wang, Y.; Miao, X.; Liu, Y.; Li, F.; Liu, Q.; Sun, J.; Cai, L. Dysregulation of histone acetyltransferases and deacetylases in cardiovascular diseases. *Oxidative medicine and cellular longevity* **2014**, *2014*, 641979.
106. Azorsa, D.O.; Robeson, R.H.; Frost, D.; Meec hoovet, B.; Brautigam, G.R.; Dickey, C.; Beaudry, C.; G.D.; Holz, D.R.; Hernandez, J.A., *et al.* High-content sirna screening of the kinome identifies kinases involved in alzheimer's disease-related tau hyperphosphorylation. *BMC genomics* **2010**, *11*, 25.
107. Cheetham, J.E.; Martzen, M.R.; Kazee, A.M.; Coleman, P.D. Gap-43 message levels in anterior cerebellum in alzheimer's disease. *Brain research. Molecular brain research* **1996**, *36*, 145-151.
108. de la Monte, S.M.; Ng, S.C.; Hsu, D.W. Aberrant gap-43 gene expression in alzheimer's disease. *The American journal of pathology* **1995**, *147*, 934-946.
109. Beurel, E.; Grieco, S.F.; Jope, R.S. Glycogen synthase kinase-3 (gsk3): Regulation, actions, and diseases. *Pharmacology & therapeutics* **2015**, *148*, 114-131.
110. Kremer, A.; Louis, J.V.; Jaworski, T.; Van Leuven, F. Gsk3 and alzheimer's disease: Facts and fiction. *Frontiers in molecular neuroscience* **2011**, *4*, 17.
111. Lal, H.; Ahmad, F.; Woodgett, J.; Force, T. The gsk-3 family as therapeutic target for myocardial diseases. *Circulation research* **2015**, *116*, 138-149.
112. Ngkelo, A.; Hoffmann, R.F.; Durham, A.L.; Marwick, J.A.; Brandenburg, S.M.; de Bruin, H.G.; Jonker, M.R.; Rossios, C.; Tsitsiou, E.; Caramori, G., *et al.* Glycogen synthase kinase-3beta modulation of glucocorticoid responsiveness in copd. *American journal of physiology. Lung cellular and molecular physiology* **2015**, *309*, L1112-1123.
113. King, G.D.; Scott Turner, R. Adaptor protein interactions: Modulators of amyloid precursor protein metabolism and alzheimer's disease risk? *Experimental neurology* **2004**, *185*, 208-219.
114. Biederer, T.; Cao, X.; Sudhof, T.C.; Liu, X. Regulation of app-dependent transcription complexes by mint/x11s: Differential functions of mint isoforms. *The Journal of neuroscience : the official journal of the Society for Neuroscience* **2002**, *22*, 7340-7351.
115. Sabo, S.L.; Lanier, L.M.; Ikin, A.F.; Khorkova, O.; Sahasrabudhe, S.; Greengard, P.; Buxbaum, J.D. Regulation of beta-amyloid secretion by fe65, an amyloid protein precursor-binding protein. *The Journal of biological chemistry* **1999**, *274*, 7952-7957.
116. Chen, Y.; Liu, W.; McPhie, D.L.; Hassinger, L.; Neve, R.L. App-bp1 mediates app-induced apoptosis and DNA synthesis and is increased in alzheimer's disease brain. *The Journal of cell biology* **2003**, *163*, 27-33.
117. Jans, D.M.; Martinet, W.; Van De Parre, T.J.; Herman, A.G.; Bult, H.; Kockx, M.M.; De Meyer, G.R. Processing of amyloid precursor protein as a biochemical link between atherosclerosis and disease. *Cardiovascular & hematological disorders drug targets* **2006**, *6*, 21-34.
118. De Meyer, G.R.; De Cleen, D.M.; Cooper, S.; Knaapen, M.W.; Jans, D.M.; Martinet, W.; Herman, A.G.; Bult, H.; Kockx, M.M. Platelet phagocytosis and processing of beta-amyloid precursor protein as a mechanism of macrophage activation in atherosclerosis. *Circulation research* **2002**, *90*, 1197-1204.
119. Stamatelopoulos, K.; Sibbing, D.; Rallidis, L.S.; Georgiopoulos, G.; Stakos, D.; Braun, S.; Gatsiou, A.; Sopova, K.; Kotakos, C.; Varounis, C., *et al.* Amyloid-beta (1-40) and the risk of death from cardiovascular causes in patients with coronary heart disease. *Journal of the American College of Cardiology* **2015**, *65*, 904-916.
120. Bu, X.L.; Cao, G.Q.; Shen, L.L.; Xiang, Y.; Jiao, S.S.; Liu, Y.H.; Zhu, C.; Zeng, F.; Wang, Q.H.; Wang, Y.R., *et al.* Serum amyloid-beta levels are increased in patients with chronic obstructive pulmonary disease. *Neurotoxicity research* **2015**, *28*, 346-351.

121. Natunen, T.; Takalo, M.; Kemppainen, S.; Leskela, S.; Marttinen, M.; Kurkinen, K.M.A.; Pursiheimo, J.P.; Sarajarvi, T.; Viswanathan, J.; Gabbouj, S., *et al.* Relationship between ubiquilin-1 and bace1 in human alzheimer's disease and apde9 transgenic mouse brain and cell-based models. *Neurobiology of disease* **2016**, *85*, 187-205.
122. Hu, C.; Tian, Y.; Xu, H.; Pan, B.; Terpstra, E.M.; Wu, P.; Wang, H.; Li, F.; Liu, J.; Wang, X. Inadequate ubiquitination-proteasome coupling contributes to myocardial ischemia-reperfusion injury. *The Journal of clinical investigation* **2018**, *128*, 5294-5306.
123. Lin, Y.Z.; Zhong, X.N.; Chen, X.; Liang, Y.; Zhang, H.; Zhu, D.L. Roundabout signaling pathway involved in the pathogenesis of copd by integrative bioinformatics analysis. *International journal of chronic obstructive pulmonary disease* **2019**, *14*, 2145-2162.
124. Serneels, L.; Van Biervliet, J.; Craessaerts, K.; Dejaegere, T.; Horre, K.; Van Houtvin, T.; Esselmann, H.; Paul, S.; Schafer, M.K.; Berezovska, O., *et al.* Gamma-secretase heterogeneity in the aph1 subunit: Relevance for alzheimer's disease. *Science* **2009**, *324*, 639-642.
125. Serneels, L.; Dejaegere, T.; Craessaerts, K.; Horre, K.; Jorissen, E.; Tousseyn, T.; Hebert, S.; Coolen, M.; Martens, G.; Zwijsen, A., *et al.* Differential contribution of the three aph1 genes to gamma-secretase activity in vivo. *Proc Natl Acad Sci U S A* **2005**, *102*, 1719-1724.
126. Kaether, C.; Haass, C.; Steiner, H. Assembly, trafficking and function of gamma-secretase. *Neuro-degenerative diseases* **2006**, *3*, 275-283.
127. Zong, D.; Ouyang, R.; Li, J.; Chen, Y.; Chen, P. Notch signaling in lung diseases: Focus on notch1 and notch3. *Therapeutic advances in respiratory disease* **2016**, *10*, 468-484.
128. Aquila, G.; Pannella, M.; Morelli, M.B.; Caliceti, C.; Fortini, C.; Rizzo, P.; Ferrari, R. The role of notch pathway in cardiovascular diseases. *Global cardiology science & practice* **2013**, *2013*, 364-371.
129. Jo, D.G.; Arumugam, T.V.; Woo, H.N.; Park, J.S.; Tang, S.C.; Mughal, M.; Hyun, D.H.; Park, J.H.; Choi, Y.H.; Gwon, A.R., *et al.* Evidence that gamma-secretase mediates oxidative stress-induced beta-secretase expression in alzheimer's disease. *Neurobiology of aging* **2010**, *31*, 917-925.
130. Asai, M.; Yagishita, S.; Iwata, N.; Saido, T.C.; Ishiura, S.; Maruyama, K. An alternative metabolic pathway of amyloid precursor protein c-terminal fragments via cathepsin b in a human neuroglioma model. *FASEB journal : official publication of the Federation of American Societies for Experimental Biology* **2011**, *25*, 3720-3730.
131. Chai, Y.L.; Chong, J.R.; Weng, J.; Howlett, D.; Halsey, A.; Lee, J.H.; Attems, J.; Aarsland, D.; Francis, P.T.; Chen, C.P., *et al.* Lysosomal cathepsin d is upregulated in alzheimer's disease neocortex and may be a marker for neurofibrillary degeneration. *Brain pathology* **2019**, *29*, 63-74.
132. Liu, C.L.; Guo, J.; Zhang, X.; Sukhova, G.K.; Libby, P.; Shi, G.P. Cysteine protease cathepsins in cardiovascular disease: From basic research to clinical trials. *Nature reviews. Cardiology* **2018**, *15*, 351-370.
133. Wang, H.; Sang, N.; Zhang, C.; Raghupathi, R.; Tanzi, R.E.; Saunders, A. Cathepsin l mediates the degradation of novel app c-terminal fragments. *Biochemistry* **2015**, *54*, 2806-2816.
134. Varma, V.R.; Varma, S.; An, Y.; Hohman, T.J.; Seddighi, S.; Casanova, R.; Beri, A.; Dammer, E.B.; Seyfried, N.T.; Pletnikova, O., *et al.* Alpha-2 macroglobulin in alzheimer's disease: A marker of neuronal injury through the rcan1 pathway. *Molecular psychiatry* **2017**, *22*, 13-23.
135. Guo, X.; Tang, P.; Li, X.; Chong, L.; Zhang, X.; Li, R. Association between two alpha-2-macroglobulin gene polymorphisms and parkinson's disease: A meta-analysis. *The International journal of neuroscience* **2016**, *126*, 193-198.

136. Yang, S.; Chen, L.; Sun, S.; Shah, P.; Yang, W.; Zhang, B.; Zhang, Z.; Chan, D.W.; Kass, D.A.; van Eyk, J.E., *et al.* Glycoproteins identified from heart failure and treatment models. *Proteomics* **2015**, *15*, 567-579.

137. Barrachina, M.N.; Calderon-Cruz, B.; Fernandez-Rocca, L.; Garcia, A. Application of extracellular vesicles proteomics to cardiovascular disease: Guidelines, data analysis, and future perspectives. *Proteomics* **2019**, *19*, e1800247.

138. Eckman, E.A.; Watson, M.; Marlow, L.; Sambamurti, K.; Eckman, C.B. Alzheimer's disease beta-amyloid peptide is increased in mice deficient in endothelin-converting enzyme. *The Journal of biological chemistry* **2003**, *278*, 2081-2084.

139. Van Uden, E.; Kang, D.E.; Koo, E.H.; Masliah, E. Ldl receptor-related protein (lrp) in alzheimer's disease: Towards a unified theory of pathogenesis. *Microscopy research and technique* **2000**, *50*, 268-272.

140. Boucher, P.; Gotthardt, M.; Li, W.P.; Anderson, R.G.; Herz, J. Lrp: Role in vascular wall integrity and protection from atherosclerosis. *Science* **2003**, *300*, 329-332.

141. Terni, B.; Ferrer, I. Abnormal expression and distribution of mmp2 at initial stages of alzheimer's disease-related pathology. *Journal of Alzheimer's disease : JAD* **2015**, *46*, 461-469.

142. Ye, S. Influence of matrix metalloproteinase genotype on cardiovascular disease susceptibility and outcome. *Cardiovascular research* **2006**, *69*, 636-645.

143. Greenlee, K.J.; Werb, Z.; Kheradmand, F. Matrix metalloproteinases in lung: Multiple, multifarious, and multifaceted. *Physiological reviews* **2007**, *87*, 69-98.

144. Stahl, B.; Diehlmann, A.; Sudhof, T.C. Direct interaction of alzheimer's disease-related presenilin 1 with armadillo protein p0071. *The Journal of biological chemistry* **1999**, *274*, 9141-9148.

145. Villarreal-Calderon, R.; Franco-Lira, M.; Gonzalez-Maciel, A.; Reynoso-Robles, R.; Harritt, L.; Perez-Guille, B.; Ferreira-Azevedo, L.; Drecktrah, D.; Zhu, H.; Sun, Q., *et al.* Up-regulation of mrna ventricular prnp prion protein gene expression in air pollution highly exposed young urbanites: Endoplasmic reticulum stress, glucose regulated protein 78, and nanosized particles. *International journal of molecular sciences* **2013**, *14*, 23471-23491.

146. Korytina, G.F.; Akhmadishina, L.Z.; Aznabaeva, Y.G.; Kochetova, O.V.; Zagidullin, N.S.; Kzhyshkowska, J.G.; Zagidullin, S.Z.; Viktorova, T.V. Associations of the nrf2/keap1 pathway and antioxidant defense gene polymorphisms with chronic obstructive pulmonary disease. *Gene* **2019**, *692*, 102-112.

147. Yoshino, Y.; Mori, T.; Yoshida, T.; Yamazaki, K.; Ozaki, Y.; Sao, T.; Funahashi, Y.; Iga, J.I.; Ueno, S.I. Elevated mrna expression and low methylation of snca in japanese alzheimer's disease subjects. *of Alzheimer's disease : JAD* **2016**, *54*, 1349-1357.

148. Rodriguez-Araujo, G.; Nakagami, H.; Takami, Y.; Katsuya, T.; Akasaka, H.; Saitoh, S.; Shimamoto, K.; Morishita, R.; Rakugi, H.; Kaneda, Y. Low alpha-synuclein levels in the blood are associated with insulin resistance. *Scientific reports* **2015**, *5*, 12081.

149. Rockenstein, E.; Hansen, L.A.; Mallory, M.; Trojanowski, J.Q.; Galasko, D.; Masliah, E. Altered expression of the synuclein family mrna in lewy body and alzheimer's disease. *Brain research* **2001**, 48-56.

150. Su, J.H.; Zhao, M.; Anderson, A.J.; Srinivasan, A.; Cotman, C.W. Activated caspase-3 expression in alzheimer's and aged control brain: Correlation with alzheimer pathology. *Brain research* **2001**, *898*, 350-357.

151. Kajiwara, Y.; McKenzie, A.; Dorr, N.; Gama Sosa, M.A.; Elder, G.; Schmeidler, J.; Dickstein, D.L.; Bozdagi, O.; Zhang, B.; Buxbaum, J.D. The human-specific casp4 gene product contributes to alzheimer-related synaptic and behavioural deficits. *Human molecular genetics* **2016**, *25*, 4315-4327.
152. Kim, N.H.; Kang, P.M. Apoptosis in cardiovascular diseases: Mechanism and clinical implications. *Korean circulation journal* **2010**, *40*, 299-305.
153. Demedts, I.K.; Demoor, T.; Bracke, K.R.; Joos, G.F.; Brusselle, G.G. Role of apoptosis in the pathogenesis of copd and pulmonary emphysema. *Respiratory research* **2006**, *7*, 53.
154. Ray, S.; Britschgi, M.; Herbert, C.; Takeda-Uchimura, Y.; Boxer, A.; Blennow, K.; Friedman, L.F.; Galasko, D.R.; Jutel, M.; Karydas, A., *et al.* Classification and prediction of clinical alzheimer's diagnosis based on plasma signaling proteins. *Nature medicine* **2007**, *13*, 1359-1362.
155. Dursun, E.; Gezen-Ak, D.; Hanagasi, H.; Bilgic, B.; Lohmann, E.; Ertan, S.; Atasoy, I.L.; Alaylioglu, M.; Araz, O.S.; Onal, B., *et al.* The interleukin 1 alpha, interleukin 1 beta, interleukin 6 and alpha-2-macroglobulin serum levels in patients with early or late onset alzheimer's disease, mild cognitive impairment or parkinson's disease. *Journal of neuroimmunology* **2015**, *283*, 50-57.
156. Abbate, A.; Toldo, S.; Marchetti, C.; Kron, J.; Van Tassell, B.W.; Dinarello, C.A. Interleukin-1 and the inflammasome as therapeutic targets in cardiovascular disease. *Circulation research* **2020**, *126*, 1260-1280.
157. Rabolli, V.; Badissi, A.A.; Devosse, R.; Uwambayinema, F.; Yakoub, Y.; Palmai-Pallag, M.; Lebrun, A.; De Gussem, V.; Couillin, I.; Ryffel, B., *et al.* The alarmin il-1alpha is a master cytokine in acute lung inflammation induced by silica micro- and nanoparticles. *Particle and fibre toxicology* **2014**, *11*, 69.
158. Pedrero-Prieto, C.M.; Flores-Cuadrado, A.; Saiz-Sanchez, D.; Ubeda-Banon, I.; Frontinan-Rubio, J.; Alcain, F.J.; Mateos-Hernandez, L.; de la Fuente, J.; Duran-Prado, M.; Villar, M., *et al.* Human amyloid-beta enriched extracts: Evaluation of in vitro and in vivo internalization and molecular characterization. *Alzheimer's research & therapy* **2019**, *11*, 56.
159. Charlesworth, J.C.; Curran, J.E.; Johnson, M.P.; Goring, H.H.; Dyer, T.D.; Diego, V.P.; Kent, J.W., Jr.; Mahaney, M.C.; Almasy, L.; MacCluer, J.W., *et al.* Transcriptomic epidemiology of smoking: The effect of smoking on gene expression in lymphocytes. *BMC medical genomics* **2010**, *3*, 29.
160. Ashford, J.W.; Soultanian, N.S.; Zhang, S.X.; Geddes, J.W. Neuropil threads are collinear with map2 immunostaining in neuronal dendrites of alzheimer brain. *Journal of neuropathology and experimental neurology* **1998**, *57*, 972-978.
161. Mercer, B.A.; Lemaitre, V.; Powell, C.A.; D'Armiento, J. The epithelial cell in lung health and emphysema pathogenesis. *Current respiratory medicine reviews* **2006**, *2*, 101-142.
162. Iqbal, K.; Liu, F.; Gong, C.X.; Grundke-Iqbal, I. Tau in alzheimer disease and related tauopathies. *Current Alzheimer research* **2010**, *7*, 656-664.
163. Fuster-Matanzo, A.; Llorens-Martin, M.; Jurado-Arjona, J.; Avila, J.; Hernandez, F. Tau protein and adult hippocampal neurogenesis. *Frontiers in neuroscience* **2012**, *6*, 104.
164. Morrow, J.D.; Cho, M.H.; Platig, J.; Zhou, X.; DeMeo, D.L.; Qiu, W.; Celli, B.; Marchetti, N.; Criner, Bueno, R., *et al.* Ensemble genomic analysis in human lung tissue identifies novel genes for chronic obstructive pulmonary disease. *Human genomics* **2018**, *12*, 1.
165. Wu, W.; Jiang, H.; Wang, M.; Zhang, D. Meta-analysis of the association between urokinase-plasminogen activator gene rs2227564 polymorphism and alzheimer's disease. *American journal of Alzheimer's disease and other dementias* **2013**, *28*, 517-523.

166. Xu, J.; Li, W.; Bao, X.; Ding, H.; Chen, J.; Zhang, W.; Sun, K.; Wang, J.; Wang, X.; Wang, H., *et al.* Association of putative functional variants in the plau gene and the plaur gene with myocardial infarction. *Clinical science* **2010**, *119*, 353-359.
167. Wang, I.M.; Stepaniants, S.; Boie, Y.; Mortimer, J.R.; Kennedy, B.; Elliott, M.; Hayashi, S.; Loy, L.; Coulter, S.; Cervino, S., *et al.* Gene expression profiling in patients with chronic obstructive pulmonary disease and lung cancer. *American journal of respiratory and critical care medicine* **2008**, *177*, 402-411.
168. Tucker, H.M.; Kihiko, M.; Caldwell, J.N.; Wright, S.; Kawarabayashi, T.; Price, D.; Walker, D.; Scheff, S.; McGillis, J.P.; Rydel, R.E., *et al.* The plasmin system is induced by and degrades amyloid-beta aggregates. *The Journal of neuroscience : the official journal of the Society for Neuroscience* **2000**, *20*, 3937-3946.
169. Caputo, M.; Mantini, G.; Floriani, I.; Ciceri, M.; Noseda, A.; Bonomo, L. Tissue plasminogen activator, tissue plasminogen activator inhibitor and lipoprotein(a) in patients with coronary, epiaortic and peripheral occlusive artery disease. *European heart journal* **1996**, *17*, 1329-1336.
170. Silveyra, M.X.; Garcia-Ayllon, M.S.; de Barreda, E.G.; Small, D.H.; Martinez, S.; Avila, J.; Saez-Valero, Altered expression of brain acetylcholinesterase in ftdp-17 human tau transgenic mice. *Neurobiology of aging* **2012**, *33*, 624 e623-634.
171. Garcia-Ayllon, M.S.; Small, D.H.; Avila, J.; Saez-Valero, J. Revisiting the role of acetylcholinesterase in alzheimer's disease: Cross-talk with p-tau and beta-amyloid. *Frontiers in molecular neuroscience* **2011**, *4*, 22.
172. Wu, P.H.; Lin, Y.T.; Hsu, P.C.; Yang, Y.H.; Lin, T.H.; Huang, C.T. Impact of acetylcholinesterase inhibitors on the occurrence of acute coronary syndrome in patients with dementia. *Scientific reports* **2015**, *5*, 15451.
173. Ben Anes, A.; Ben Nasr, H.; Garrouch, A.; Bennour, S.; Bchir, S.; Hachana, M.; Benzarti, M.; Tabka, Z.; Chahed, K. Alterations in acetylcholinesterase and butyrylcholinesterase activities in chronic obstructive pulmonary disease: Relationships with oxidative and inflammatory markers. *Molecular cellular biochemistry* **2018**, *445*, 1-11.
174. Lin, H.Q.; Wang, Y.; Chan, K.L.; Ip, T.M.; Wan, C.C. Differential regulation of lipid metabolism genes in the brain of acetylcholinesterase knockout mice. *Journal of molecular neuroscience : MN* **2014**, *53*, 397-408.
175. Beeri, M.S.; Sonnen, J. Brain bdnf expression as a biomarker for cognitive reserve against alzheimer disease progression. *Neurology* **2016**, *86*, 702-703.
176. Song, J.H.; Yu, J.T.; Tan, L. Brain-derived neurotrophic factor in alzheimer's disease: Risk, and therapy. *Molecular neurobiology* **2015**, *52*, 1477-1493.
177. Numakawa, T.; Suzuki, S.; Kumamaru, E.; Adachi, N.; Richards, M.; Kunugi, H. Bdnf function and intracellular signaling in neurons. *Histology and histopathology* **2010**, *25*, 237-258.
178. Hacioglu, G.; Senturk, A.; Ince, I.; Alver, A. Assessment of oxidative stress parameters of neurotrophic factor heterozygous mice in acute stress model. *Iranian journal of basic medical sciences* **2016**, *19*, 388-393.
179. de Araujo, C.L.P.; da Silva, I.R.V.; Reinaldo, G.P.; Peccin, P.K.; Pochmann, D.; Teixeira, P.J.Z.; Elsner, V.R.; Dal Lago, P. Pulmonary rehabilitation and bdnf levels in patients with chronic obstructive pulmonary disease: A pilot study. *Respiratory physiology & neurobiology* **2019**, *259*, 63-69.
180. Sustar, A.; Perkovic, M.N.; Erjavec, G.N.; Strac, D.S.; Pivac, N. Association between reduced brain-derived neurotrophic factor concentration & coronary heart disease. *The Indian journal of medical research* **2019**, *150*, 43-49.
